# Genome‐wide screening of potential RNase Y‐processed mRNAs in the M49 serotype *Streptococcus pyogenes* NZ131

**DOI:** 10.1002/mbo3.671

**Published:** 2018-06-13

**Authors:** Zhiyun Chen, Rahul Raghavan, Fengxia Qi, Justin Merritt, Jens Kreth

**Affiliations:** ^1^ Department of Microbiology and Immunology University of Oklahoma Health Sciences Center Oklahoma City Oklahoma; ^2^ Department of Biology and Center for Life in Extreme Environments Portland State University Portland Oregon; ^3^ Department of Restorative Dentistry Oregon Health and Science University Portland Oregon; ^4^ Department of Molecular Microbiology and Immunology Oregon Health and Science University Portland Oregon

**Keywords:** genomics, RNA processing, RNase Y, *Streptococcus pyogenes*

## Abstract

RNase Y is a major endoribonuclease in Group A streptococcus (GAS) and other Gram‐positive bacteria. Our previous study showed that RNase Y was involved in mRNA degradation and processing in GAS. We hypothesized that mRNA processing regulated the expression of important GAS virulence factors via altering their mRNA stabilities and that RNase Y mediated at least some of the mRNA‐processing events. The aims of this study were to (1) identify mRNAs that were processed by RNase Y and (2) confirm the mRNA‐processing events. The transcriptomes of *Streptococcus pyogenes* NZ131 wild type and its RNase Y mutant (*Δrny*) were examined with RNA‐seq. The data were further analyzed to define GAS operons. The mRNA stabilities of the wild type and *Δrny* at subgene level were determined with tiling array analysis. Operons displaying segmental stability in the wild type but not in the *Δrny* were predicted to be RNase Y processed. Overall 865 operons were defined and their boundaries predicted. Further analysis narrowed down 15 mRNAs potentially processed by RNase Y. A selection of four candidates including *folC1* (folylpolyglutamate synthetase), *prtF* (fibronectin‐binding protein), *speG* (streptococcal exotoxin G), *ropB* (transcriptional regulator of *speB*), and *ypaA* (riboflavin transporter) mRNAs was examined with Northern blot analysis. However, only *folC1* was confirmed to be processed, but it is unlikely that RNase Y is responsible. We conclude that GAS use RNase Y to selectively process mRNA, but the overall impact is confined to selected virulence factors.

## 1 INTRODUCTION

RNA processing, also known as RNA maturation or posttranscriptional modification, is a process that converts primary transcript RNA into mature RNA. RNA maturation is common in eukaryotes requiring extensive processing of the primary transcripts (Lodish et al., 2000; Nazar, 2004). Prokaryotic RNA processing has been reported mainly in stable RNAs belonging to the protein synthesis machinery (rRNAs and tRNAs), but rarely in mRNAs or small RNAs (Condon, 2007; Deutscher, 2006). However, emerging evidence indicates that bacterial RNA processing also takes place in mRNAs and small RNAs and plays an important role in gene regulation.

Processing of a polycistronic mRNA leads to differential stability of individual cistrons, which allows cistrons in the same operon to be expressed at different levels. Known examples in *Bacillus subtilis* include the *dnaK* operon, the *gapA* operon, and the *ilv‐leu* operon (Bechhofer, 2009). This endonucleolytic processing alters the stability of individual cistrons residing in these polycistronic mRNAs to reduce the stability of segments encoding proteins needed in lower abundance and increasing the stability of segments encoding proteins needed in higher abundance (Ludwig, Rebhan, Blencke, Merzbacher, & Stulke, 2002; Ludwig et al., 2001; Meinken, Blencke, Ludwig, & Stulke, 2003). RNA processing is also necessary for maturation and functional activity of some noncoding small RNAs. For example, *Streptococcus pyogenes* CRISPR RNA, which is important in the interference with foreign genetic elements like plasmids and phages, must be cleaved within the repeat sequences by RNase III to generate the active spacer RNA (Deltcheva et al., 2011).

Processing in the untranslated region (UTR) of a monocistronic mRNA can change the stability of the mRNA molecule, making the processed transcript more or less stable than the primary transcript (Condon & Bechhofer, 2011). Previously, we have investigated the posttranscriptional regulation of *speB*, a gene that encodes an extracellular protease secreted by *S. pyogenes* in late exponential and stationary phases. Our transcript analyses demonstrated that the primary *speB* transcript was modified via endonuclease cleavage in the 5’ UTR, and that the processed *speB* transcript was threefold more stable than the primary transcript. The *ropB* mRNA, which encodes a transcriptional activator of *speB* and is divergently transcribed from the same intergenic region as *speB*, is also modified in the 5’ UTR in a similar manner and becomes more stable after the modification (Chen, Itzek, Malke, Ferretti, & Kreth, 2013). Interestingly, both processing events were mediated by RNase Y, a major *S. pyogenes* endoribonuclease (Chen, Itzek, Malke, Ferretti, & Kreth, 2012; Chen et al., 2013). These observations led us to suspect that mRNA processing might occur more frequently in *S. pyogenes* than was generally assumed and that RNase Y might play a major role in this process.

Here, we used a combination of RNA‐seq, tiling microarray and Northern blot analysis to identify processed mRNAs in the *S. pyogenes* genome and to further investigate the role of RNase Y in mRNA processing. RNA‐seq was used to predict *S. pyogenes* operons and to define operon boundaries with high resolution. Tiling arrays were then used to calculate mRNA half‐lives across entire transcripts to identify those with altered segmental stabilities (regions of different RNA stability relative to the rest of the transcript). Transcripts displaying altered segmental stabilities in the wild type (WT) but not in the RNase Y mutant were identified as candidate RNase Y‐processed mRNAs. Selected transcripts were then further examined to confirm RNA processing and to determine its effect upon protein production.

## 2 METHODS

### 2.1 Bacterial strains and growth conditions

*Streptococcus pyogenes* NZ131 (M49) (McShan et al., 2008) and its derivatives were routinely grown in C medium (0.5% proteose peptone #3; 1.5% yeast extract; 10 mM K_2_HPO_4_; 0.4 mM MgSO_4_; 17 mM NaCl) (Lyon, Gibson, & Caparon, 1998) at 37°C without aeration. Erythromycin and spectinomycin were added at a final concentration of 2 μg/ml and 150 μg/ml when required. The deletion of *rny* was accomplished by an overlap extension PCR technique replacing *rny* with an erythromycin resistance cassette. The RNase Y gene deleted strain (Δ*rny*), its complement strain (Δ*rny_*pDL278::rny), and Δ*rny* carrying an empty vector (Δ*rny*_pDL278) were reported in a previous study (Chen et al., 2012).

### 2.2 RNA‐seq sample preparation and sequencing

RNA extraction and purification were carried out by following a previously described protocol (Ajdic & Pham, 2007; Chen et al., 2012). Briefly, total RNA was extracted from bacterial samples with TRIzol reagent (Invitrogen, Carlsbad, CA) and was treated twice with Turbo DNase (Invitrogen) to remove chromosomal DNA. Ribosomal RNAs were removed from the total RNA by using a bacteria‐specific rRNA‐depletion kit (Ribo‐Zero, Illumina). RNA integrity before and after the treatment was determined on an RNA Nano LabChip (Agilent). RNA samples were sent to the Oklahoma Medical Research Foundation (OMRF, Oklahoma City, OK) for cDNA library construction using TruSeq RNA Sample Preparation v2 Protocol (Illumina) and DNA sequencing (100 bp paired‐end) on Illumina HiSeq 2000.

### 2.3 Alignment of sequencing reads to genome

*Streptococcus pyogenes* NZ131 complete genome was obtained from NCBI (accession No. CP000829). Bowtie2 (Langmead & Salzberg, 2012) was used to generate the *S. pyogenes* genome index and to align paired‐end RNA‐seq data using “very‐sensitive‐local” settings. The aligned files were sorted, converted to BAM format, and indexed using Samtools (Li et al., 2009). The locations of genes and tRNAs were determined by aligning corresponding FASTA files to the *S. pyogenes* genome and converting the aligned sequences into BED format. Percentage of unambiguously mapped reads was determined with CLC Genomics Workbench (Qiagen).

### 2.4 Reads coverage depth calculation

Reads coverage depth of each base on the chromosome (called “base read” in the following sections) was computed with BEDTools using the “genomeCoverageBed” function (Quinlan & Hall, 2010). The base reads were log2 transformed to reduce in‐gene variation. To calculate the expression level of a particular sequence, the log‐transformed base reads in that sequence were added, averaged, and exponentially transformed.

### 2.5 Operon prediction

We used the following criteria to determine whether two consecutive genes belonged to the same operon: (i) they were no more than 100 bp apart on the chromosome; (ii) genes were encoded on the same strand; (iii) their expression levels were no more than fourfold different; and (iv) their intergenic region did not contain a poorly expressed domain (i.e., its expression level was no more than half of the least expressed gene). A Python‐based script was developed to group *S. pyogenes* genes into operons based on these criteria. The Python code has been uploaded and can be accessed by the following link: https://github.com/ZhiyunChen/RNA_Processing.

### 2.6 Validation of operon prediction

The accuracy of the operon predictions was evaluated by comparing them to the Prokaryotic Operon DataBase (ProOpDB), a computer‐based operon prediction tool (Taboada, Ciria, Martinez‐Guerrero, & Merino, 2012).

### 2.7 Operon boundary identification

We assumed that base reads within a gene transcript should be stable but would drastically increase or decrease at the border. Based on this assumption, we used the coefficient of variation (CV) of base reads as a key indicator for transcription boundary identification. To identify the transcriptional start site (TSS) of an operon, we first defined a “search region” that covered the translational start site of the first gene in the operon and its surrounding sequences. The “search region” included the first half portion of the gene’s ORF (or the first 200 base, whichever was shorter) and extended upstream into its intergenic region until it reached the site with the lowest base read in the intergenic region. CV of all base reads in the “search region” was calculated and used as a reference (refCV). Then, a 25‐bp window was created and slid through the “search region” with a one‐base step size. CV of base reads covered by the sliding window was determined after each step. The window location displaying the maximum CV (maxCV) was considered to contain the TSS if maxCV was twofold higher than refCV. TSS was defined as the site with the lowest base read in that window. The transcriptional stop site of an operon was defined in a similar manner. To unify operon boundaries obtained from different RNA‐seq datasets, the boundary that gave the shortest UTR was selected and was regarded as the “real” boundary of that operon. The procedures mentioned above were implemented with Python scripts.

### 2.8 mRNA half‐life calculation at a subgene level

In a previous study, we used Affymetrix microarray analysis to determine *S. pyogenes* mRNA half‐lives at a single gene level (Chen et al., 2013). Since Affymetrix used multiple 25‐base oligonucleotide probes to detect one gene at different sites, it was possible to determine mRNA half‐lives at a subgene level. The chip was designed to detect the expression of each ORF with a set of 17 probes. It also detected each intergenic region with a set of tiled probes, that is, the probes were spaced at a 14‐ to 23‐base distance so that their ends overlapped. Affymetrix Power Tools were used to extract raw probe hybridization signals from microarray CEL files (GEO accession number: GSE40198). mRNA decay rate at each probe‐binding site was calculated by using a “steepest slope” method (Chen et al., 2013). The overall mRNA half‐life of an operon was obtained as an averaged half‐life of all probe‐binding sites that belonged to the operon.

### 2.9 RNase Y‐processed mRNA identification

We reasoned that an unprocessed transcript would have a uniform decay rate throughout its sequence in both WT and ∆*rny* strains, but a transcript that is processed by RNase Y would display altered segmental stability in WT in comparison to the ∆*rny* strain (Mader, Hennig, Hecker, & Homuth, 2004). We screened the *S. pyogenes* transcriptome for operons displaying segmental RNA stabilities. An operon was considered to represent a potentially processed transcript if it contained a domain in which its average half‐life was twofold higher or lower than the overall operon half‐life. To reduce false positives, we mandatorily defined that the domain must contain at least two consecutive probe‐binding sites. Two lists of processed RNA candidates in the WT and ∆*rny* were generated, respectively. The lists were compared to find operons that were present in the WT but absent in the ∆*rny*. These operons were likely processed by RNase Y specifically. The procedures mentioned above were implemented with Python scripts.

### 2.10 Northern blot, real‐time PCR, and 5’ RACE analyses

Northern blot, real‐time‐PCR, and 5’ RACE analyses were carried out as described previously (Chen et al., 2012, 2013). Additionally, for Northern blot analysis of the FMN riboswitch 5S rRNA was used as loading control to account for the small size of the transcript. The Northern blot probe sequences are listed in Table 1.

**Table 1:** Characteristics of Northern blot probes

Name	Sequence	Gene locus	Orientation on chromosome	Start point	Amplicon (bp)
folC1_1F	AAGGGAGTGCATATCGTTGG	Spy49_0851	Forward	851069	434
folC1_1R(T7)	taa tac gac tca cta tag gg GCGGCAATATCAGCGTAAGT	Spy49_0851	Reverse	851502	
folC1_2F	cacatcgctatgccaggac	Spy49_0851	Forward	851696	400
folC1_2R(T7)	taa tac gac tca cta tag gg tgaaatcagcaaccctacca	Spy49_0851	Reverse	852095	
speG_5’UTR_1L	gcaacacttgtgcgtgaagt	Spy49_ig0084	Forward	194721	152
speG_5’UTR_1R(T7)	taa tac gac tca cta tag gg cctaaataaaaatatcaatcggtttca	Spy49_ig0084	Reverse	194872	
speG_1F	ACCCCATGCGATTATGAAAA	Spy49_0187	Forward	195031	310
speG_1R(T7)	taa tac gac tca cta tag gg GAACAACCTCAGAGGGCAAA	Spy49_0187	Reverse	195340	
ypaA_LD_1L	cgagcgcaagctgatgtg	Spy49_ig0086	Forward	319649	120
ypaA_LD_1R(T7)	taa tac gac tca cta tag gg aaattgaaagaagttccgtcgt	Spy49_ig0086	Reverse	319768	
ypaA_1F	ATTATTCCAGGCGCAGCTTT	Spy49_0307	Forward	319870	426
ypaA_1R(T7)	taa tac gac tca cta tag gg GCAAATATCAACCCCCTCAA	Spy49_0307	Reverse	320295	
prtF_5’UTR_1L	TTT GAC AGT TGT CCT GTA GTC TTT	Spy49_ig0045	Forward	125913	79
prtF_5’UTR_1R(T7)	TAA TAC GAC TCA CTA TAG GGT TGT GTC ATT TAT TTT CTC TCT CCA	Spy49_ig0045	Reverse	125991	
prtF_1F	GCTTCCGCTAGAATCAGGTG	Spy49_0119	Forward	128524	475
prtF_1R(T7)	taa tac gac tca cta tag gg TTGCTCGTCTGGAAGCTTTT	Spy49_0119	Reverse	128998	

### 2.11 Protein sample preparation and Western blot analysis

*Streptococcus pyogenes* whole‐cell lysate was prepared by incubating 10^8^ PBS‐washed cells with 2 μg streptococcal phage lysin (PlyC, kindly provided by Dr. Vincent Fischetti, Rockefeller University, New York) (Nelson, Schuch, Chahales, Zhu, & Fischetti, 2006) in a 0.1 ml reaction mix at 37°C for 10 min or until the cells completely lysed. Extracellular protein was prepared by following a TCA precipitation protocol. Briefly, 10 ml S*. pyogenes* culture was centrifuged at 5,000 ***g*** for 10 min and filtered though a 0.22 μm syringe filter (VWR). Clarified culture supernatant, after chilled on ice, was mixed with 10% volume ice cold trichloroacetic acid (TCA, 100% in acetone) and was incubated at 4°C for 3 hr. The mixture was centrifuged at 20,000***g*** at 4°C for 10 min. The pellet was washed with 1 ml ice‐cold acetone for three times to remove residual TCA. Washed pellet was briefly air‐dried and suspended in 0.1 ml H_2_O. Western blot analysis was carried out by following a previously described protocol (Kreth, Chen, Ferretti, & Malke, 2011).

### 2.12 Accession numbers

The RNA‐seq sequencing and Affymetrix microarray data have been deposited in the GEO database under accession numbers: GSE40198 and GSE99533.

## 3 RESULTS

### 3.1 Transcription map generation with RNA‐seq

mRNA processing often occurs in the 5’ UTR of a transcript (e.g., *speB* and *ropB* in *S. pyogenes*) or between ORFs of a polycistronic operon (e.g., the *gapA* and *dnaK* operons in *B. subtilis*). In order to detect these processing events, it is first necessary to have a transcriptome map that identifies the boundary of each transcript as well as the organization of each polycistronic operon. We used RNA‐seq analysis to generate a transcriptome map of nephritogenic *S. pyogenes* NZ131, serotype M49 WT, and an isogenic ∆*rny* mutant. Two independent WT and ∆*rny* mutant strains were grown in C medium until the late exponential phase and used for the construction of cDNA libraries followed by deep sequencing. Sequencing generated a total of 11–12 billion reads for each sample. Subsequently, the sequence reads were aligned to the *S. pyogenes* NZ131 genome.

### 3.2 Quality control of RNA‐seq results

**Figure 1:**
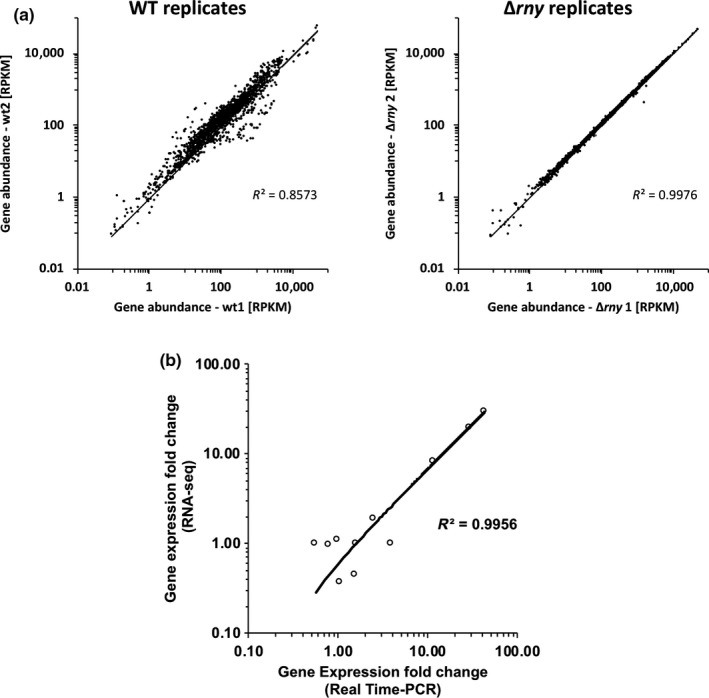
RNA‐seq quality control. (a) Consistency of gene abundance between biological replicates of *Streptococcus pyogenes* NZ131 wild type and isogenic Δ*rny* mutant determined by RNA‐seq. The gene abundance of the replicates was plotted against each other on a logarithmic scale scatter diagram. The trend lines are presented for visual determination of consistency along with calculated R^2^‐values. (b) Correlation of gene expression levels between selected genes (*gyrA, acpA, cfa, emm49, pnpA, prtF, ropB, sagA, ska, speG*, and Spy49_1276) comparing RNA‐seq and qRT‐PCR results. The trend line is presented for visual determination of consistency along with calculated *R* ^2^‐value

Among all RNA‐seq reads, more than 99% reads were unambiguously mapped to the *S. pyogenes* genome (Table S1). Among these mapped reads, less than 1% were mapped to rRNAs or tRNAs, which indicated a successful removal of structural RNAs in sample preparation (data not shown). The two biological replicates (referred to as wt1 and wt2 for the wild type and *Δrny1* and *Δrny2* for the RNase Y mutant) yielded highly consistent results (*r* ^2^ = 0.857 for the WT and *r* ^2^ = 0.998 for the Δ*rny*) (Figure 1a). Quantitative RT‐PCR (qPCR) was used to evaluate the consistency of RNA‐seq results. Eleven genes were randomly selected and their relative transcript abundance in the Δ*rny* mutant versus the wild type was determined with qRT‐PCR and RNA‐seq analysis, respectively. Results obtained from the two methods showed a strong correlation (*r* ^2^ = 0.996) (Figure 1b). We conclude that our RNA‐seq datasets are of high quality and can be used for downstream data analysis.

### 3.3 Operon prediction and transcriptome map construction

The computer‐based operon prediction tool Prokaryotic Operon DataBase (ProOpDB) was used to determine the potential number of operons encoded in the *S. pyogenes* genome (Taboada et al., 2012). This analysis was performed to be able to compare our prediction approach using RNA‐seq data to an independent method. Overall, 720 gene pairs were reported to be cotranscribed by ProOpDB.

**Figure 2:**
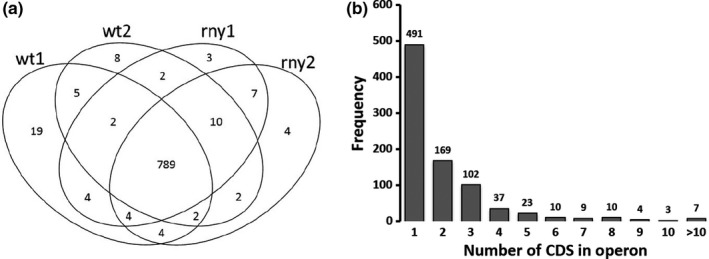
Transcriptome analysis and operon prediction. (a) Venn diagram showing the relationship of individual operon predictions using *Streptococcus pyogenes* NZ131 wild type (wt1 and wt2) and isogenic Δ*rny* mutant (rny1 and rny2) RNA‐seq datasets. (b) Distribution of the number of CDS (coding sequences) in operons in the *S. pyogenes* NZ131 genome

The first step of constructing a transcriptome map from our RNA‐seq data was to group genes into operons. *S. pyogenes* NZ131 has a single circular DNA of 1.8 million bases including 1699 protein‐encoding genes, 18 rRNAs, and 66 tRNAs (McShan et al., 2008). RNA‐seq datasets of both the WT and Δ*rny* strains were used for operon prediction and yielded highly consistent results. Specifically, predictions from WT2, Δ*rny*1, and Δ*rny*2 datasets were almost identical with only 1–2 uniquely predicted gene pairs found in each sample. Predictions from WT1 were slightly different from the others with 14 uniquely predicted gene pairs (Figure 2a). This observation suggests that an RNase Y mutation does not affect *S. pyogenes* operon organization. We, therefore, combined the four predictions to include all possible operons. A total of 865 operons were predicted, in which 491 CDS (coding sequences) were monocistronic, 169 dicistronic, 102 tricistronic, and 103 contain ≥4 CDS (Figure 2b). The Spy49_1460–Spy49_1481 operon contains the highest number of genes (20) and encodes phage proteins, while the second largest operon consists of 16 genes and encodes tRNAs. Our global operon prediction results can be found in Supplemental Table S2. Comparing our RNA‐seq analysis with the ProOpDB prediction (which we assumed to be 100% correct) found concordance in 720 cotranscribed gene pairs (81% sensitivity) and 627 monocistronic gene pairs (71% specificity). A total of 144 gene pairs were predicted as cotranscribed by RNA‐seq analysis, but not by ProOpDB. Therefore, to test their coexpression patterns, we randomly picked 10 gene pairs and indeed confirmed their cotranscription with RT‐PCR (Figure S1). We, therefore, concluded that our operon prediction strategy is highly reliable and possibly even more accurate than that of ProOpBD predictions.

### 3.4 Identification of operon boundries

The next step was to define the boundaries of each operon. The boundary of an operon was defined as an abrupt upshift or downshift of RNA‐seq reads along the chromosome. Although the transcription start and end sites (TSSs and TESs) of most genes are located outside of their coding regions, some occur within their coding regions (Remmele et al., 2014). Therefore, a “sliding‐window” method was developed to search for abrupt changes in RNA‐seq reads in the upstream and downstream intergenic regions as well as within the coding regions of each operon (see Materials and Methods for details). We screened the upstream and 5’ regions of each operon with a 25‐bp window where an abrupt increase of RNA‐seq reads occurred and to define the left operon boundary as the site where the lowest RNA‐seq read was found in that window. The same method was used to identify the right operon boundary. RNA‐seq datasets of both the WT and Δ*rny* strains were used for boundary definition and yielded similar results. Of all predicted boundaries, 1471 (85%) showed less than 10‐bp difference. We next combined the four prediction results by selecting the prediction that yielded the shortest UTR as the “true” boundary (Supplemental table S2).

**Figure 3:**
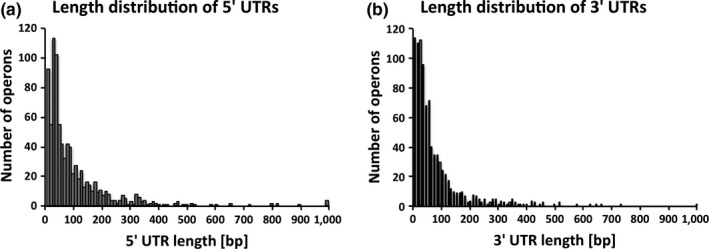
Distribution of the UTR lengths in the *Streptococcus pyogenes* NZ131 primary transcripts. (a) Length distribution of 5’ UTR. (b) Length distribution of 3’ UTR

Based on this transcriptome map, we calculated the length of UTRs for the 865 operons. The majority of 5’ UTRs varied between 0 and 200 nt with a median of 43 nt (Figure 3a). The majority of 3’ UTRs varied between 0 and 150 nt with a median of 33 nt (Figure 3b). No expression difference was observed between the WT and Δ*rny* strains, which confirmed our previous finding that the mutation of RNase Y does not affect gene expression (Chen et al., 2013).

**Figure 4:**
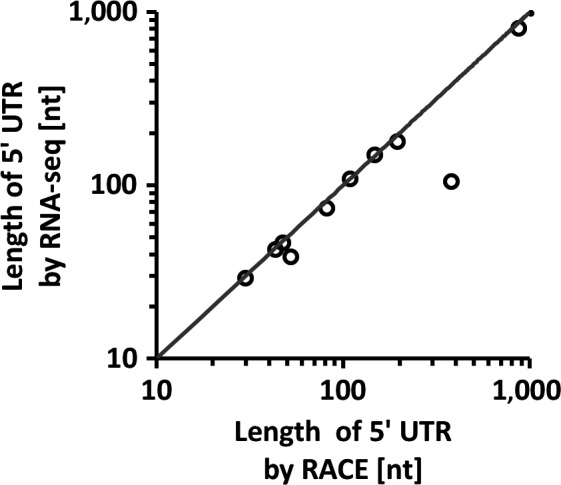
Validation of predicted 5’ UTR lengths. The lengths of 10 selected genes determined with RNA‐seq analysis was validated by 5’ RACE and the results plotted against each other on a logarithmic scale scatter diagram. The trend line is presented for visual determination of consistency. The data point outside the trend line represents the UTR of *speB* with 693nt in length

We then performed 5’ RACE on 10 highly expressed genes to test the accuracy of our transcriptional boundary predictions using RNA‐seq. Transcriptional start sites determined by the two methods were very similar to each other (Figure 4). For 6 of 10 genes, the length of the 5’ UTR as predicted by RNA‐seq was within 6 nt of the TSS identified by RACE. For three other genes, the difference was 11–12 nt. The largest discrepancy was observed for the *ropB* gene (Spy49_1691). Its 5’ UTR was determined to be 369 nt by RACE and 108 nt with RNA‐seq. A closer observation indicated that the *ropB* 5’ UTR was overlapped by its upstream gene, *speB*. When the *speB* gene was highly expressed, it apparently masked a large portion of the *ropB* 5’ UTR. We conclude that our transcriptional boundary predictions are reliable for well‐separated operons but are less reliable for overlapping operons.

### 3.5 Screen for putative RNase Y‐processed mRNAs

We screened the *S. pyogenes* transcriptome for putative RNase Y‐processed mRNAs by searching for transcripts that displayed segmental stability in the WT, but not in the Δ*rny* mutant. Segmental stability was defined as an uneven decay pattern of a transcript (i.e., the transcript contained a segment with a significantly higher or lower stability than the rest of the transcript). A Python‐based script was developed to automatically identify mRNAs with segmental stabilities in the *S. pyogenes* transcriptome. A total of 80 mRNAs displaying segmental stabilities were identified in the WT. Among these mRNAs, 29 exhibited altered diminished segmental stabilities in the ∆*rny* mutant. Visual inspection of the decay patterns of these mRNAs showed that the apparent‐altered segmental stabilities in 14 mRNAs were due to random signal variations. The remaining 15 mRNAs and their gene arrangements are presented in Table 2. Four mRNAs with a potential role in GAS virulence, *ypaA*,* speG*,* prtF*, and *folC1* were selected for further investigation. Each of these mRNAs displayed altered segmental stabilities in their 5’ regions in the WT, but not in the Δ*rny* mutant. They also showed significantly increased abundance in the Δ*rny* mutant. Northern blot analysis was carried out to examine their transcript patterns in the WT, *Δrny* mutant, *Δrny* mutant carrying an empty vector (Δ*rny*_pDL278), and complemented Δ*rny* mutant (Δ*rny*_pDL278::*rny*) strains. Results showed that the WT and Δ*rny* complemented strains yielded similar results, while the Δ*rny* and Δ*rny*_pDL278 strains yielded similar results for all examined mRNAs. These observations confirmed that the changes in transcript patterns were due to the RNase Y mutation, but not to polar effects or unknown mutations. We, therefore, report only the results from the WT and Δ*rny* mutant in the following sections.

**Table 2:** Putative RNase Y‐processed mRNAs

Operon	Strand	Gene locus	Gene name	Product name
operon_47	+	Spy49_0086	*pbp1b*	Multimodular transpeptidase‐transglycosylase
operon_62	+	Spy49_0119	*prtF*	Fibronectin‐binding protein
operon_95	+	Spy49_0187	*speG*	Exotoxin‐type G precursor
operon_105	+	Spy49_0204	*fasB*	Putative histidine kinase
		Spy49_0205	*–*	Putative histidine kinase
		Spy49_0206	*fasA*	Response regulator FasA
operon_153	+	Spy49_0307	*ypaA*	Riboflavin transporter YpaA
operon_163	+	Spy49_0325	*–*	Hypothetical protein Spy49_0325
operon_179	−	Spy49_0350c	*–*	Hypothetical protein Spy49_0350c
operon_209	+	Spy49_0418	*–*	Hypothetical protein Spy49_0418
		Spy49_0419	*–*	Putative multidrug resistance efflux pump
		Spy49_0420	*rpmG*	50S ribosomal protein L33
operon_216	+	Spy49_0433	*–*	Putative sugar transferase
		Spy49_0434	*–*	Putative glucosyl transferase
operon_290	+	Spy49_0590	*–*	Hypothetical protein Spy49_0590
		Spy49_0591	*murA*	UDP‐N‐acetylglucosamine 1‐carboxyvinyltransferase
		Spy49_0592	*–*	Hypothetical protein Spy49_0592
operon_392	+	Spy49_0851	*folC1*	Dihydrofolate synthase/folylpolyglutamate synthase
		Spy49_0852	*folE*	GTP cyclohydrolase I
		Spy49_0853	*folP*	Dihydropteroate synthase
		Spy49_0854	*folQ*	Dihydroneopterin aldolase
		Spy49_0855	*folK*	2‐amino‐4‐hydroxy‐6‐hydroxymethyldihydropteridine pyrophosphokinase
operon_448	−	Spy49_0974c	*–*	Putative lipoprotein
		Spy49_0975c	*cdd*	Cytidine deaminase
operon_463	+	Spy49_1008	*–*	Putative amino acid symporter
		Spy49_1009	*–*	Putative cation efflux system protein
operon_791	−	Spy49_1689c	*spi*	Spi SpeB protease inhibitor
		Spy49_1690c	*speB*	Strepotococcal cysteine protease (streptopain)/streptococcal pyrogenic exotoxin B (SpeB)
operon_842	+	Spy49_1781	–	Hypothetical protein Spy49_1781
		Spy49_1782	–	Hypothetical protein Spy49_1782
		Spy49_1783	–	Hypothetical protein Spy49_1783

### 3.6 FolC1

**Figure 5:**
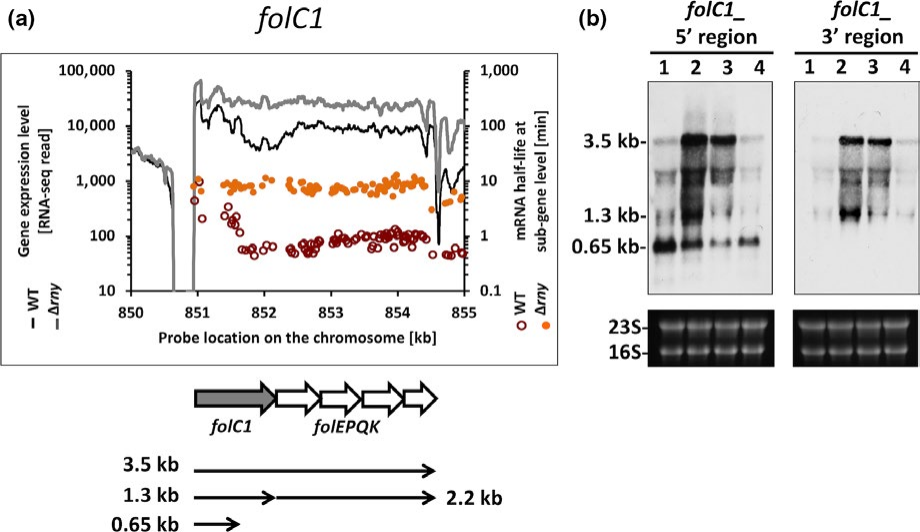
Role of RNase Y in transcript processing of *folC1*. (a) Analysis of *folC1*gene expression levels and mRNA half‐lives in *Streptococcus pyogenes* NZ131 wild type and Δ*rny* mutant. Gene expression is presented as RNA‐seq reads and mRNA half‐life is presented as probe signal intensity relative to the chromosomal position of the folate synthesis operon. (b) Northern blot analysis of the 5’ and 3’ UTRs of *folC1*. The transcripts were detected with specific probes in (1) wild type, (2) *Δrny*, (3) *Δrny* carrying an empty vector (Δ*rny*_pDL278), and (4) Δ*rny* complemented (Δ*rny*_pDL278::*rny*) strains

The *folC1* gene encodes dihydrofolate/folylpolyglutamate synthase (DHFS/FPGS), which is involved in folate synthesis and intracellular retention (Bognar, Osborne, & Shane, 1987). Operon prediction suggested that the *folC1* gene was cotranscribed with four other folate synthesis genes, *folEPQK*. mRNA stability analysis revealed segmental stabilities within the *folC1* ORF in the WT. Specifically, the 5’ region of the *folC1* gene had a measured half‐life of 2.6 min versus 0.7 min for the remaining portion of the operon. The whole operon was strongly stabilized in the ∆*rny* background (half‐life = 6.8 min) with diminished segmental stabilities (Figure 5a). Northern blot analysis of *folC1* using a probe targeting the 5’ region of the gene identified at least three transcripts with distinct sizes: 3.5 kb, 1.3 kb, and 0.65 kb. The 3.5 kb and 1.3 kb transcripts represented the *folC1‐EPQK* mRNA and the full‐length *folC1* mRNA, respectively (Figure 5b). The 0.65 kb transcript could be detected with the probe targeting the 5’ region of the gene (*folC 1.1*), but not with the probe targeting the 3’ region of the gene (*folC 1.2*). This suggested that the 0.65 kb transcript encompassed the first half of the *folC1* gene. What is the source of this truncated transcript? It seems unlikely to be created via premature transcriptional termination since no terminator‐like sequence could be identified within the *folC1* ORF. It is possible that the full‐length *folC1* transcript was processed internally so that the 3’ region of the transcript was rapidly degraded, though the 5’ region was stabilized via an unknown mechanism. However, the 0.65 kb transcript is also present in the ∆*rny* background, excluding a direct role of RNase Y in the generation of this fragment.

### 3.7 SpeG

**Figure 6:**
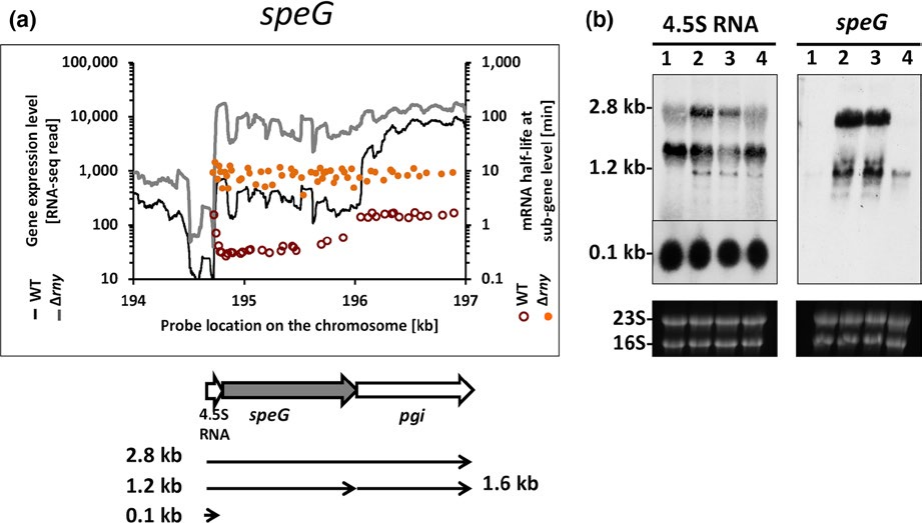
Role of RNase Y in transcript processing of *speG*. (a) Analysis of gene expression levels and mRNA half‐lives of *speG* in *Streptococcus pyogenes* NZ131 wild type and Δ*rny* mutant. Gene expression is presented as RNA‐seq reads and mRNA half‐life is presented as probe signal intensity relative to the chromosomal position of the *speG*‐encoding operon. (b) Northern blot analysis of *speG* and the upstream 4.5S RNA. The transcripts were detected with specific probes in (1) wild type, (2) *Δrny*, (3) *Δrny* carrying an empty vector (Δ*rny*_pDL278), and (4) Δ*rny* complemented (Δ*rny*_pDL278::*rny*) strains

The *speG* gene encodes superantigen G, a secreted protein that activates T cells in a nonantigen‐specific fashion and is responsible for the clinical features of streptococcal toxic shock syndrome (STSS) (Steer, Lamagni, Curtis, & Carapetis, 2012). The *speG* gene is preceded by the 4.5S RNA‐encoding gene (the RNA component of the signal recognition particle (SRP) (Herskovits, Bochkareva, & Bibi, 2000)) and followed by the *pgi* gene (encoding glucose‐6‐phosphate isomerase) on the chromosome. Sequence analysis revealed that the three genes have independent promoters (Figure 6b). Northern blot signals were barely detectable for the *speG* gene in the WT (Figure 6b). In the ∆*rny* strain, it was cotranscribed with the 4.5S RNA and *pgi* gene so that the read through transcripts (2.8 kb and 1.2 kb) became detectable. The 4.5S RNA‐encoding gene was strongly expressed in both WT and ∆*rny* mutant as a single 0.1 kb transcript (Figure 6b). The 0.1 kb transcript was unlikely the RNase Y‐processed product of mRNA read through, since the RNase Y mutation did not lead to a reduction of 4.5S RNA in abundance. It is also possible that the increased 5’ stability of the *speG* gene was due to the highly structured 4.5S RNA preceding the gene, rather than mRNA processing.

### 3.8 YpaA

**Figure 7:**
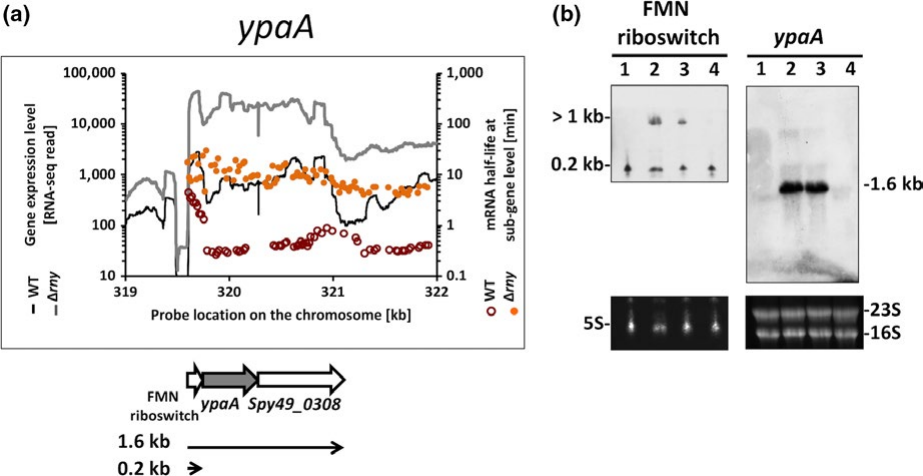
Role of RNase Y in transcript processing of *ypaA*. (a) Analysis of gene expression levels and mRNA half‐lives of *ypaA* in *Streptococcus pyogenes* NZ131 wild type and Δ*rny* mutant. Gene expression is presented as RNA‐seq reads and mRNA half‐life is presented as probe signal intensity relative to the chromosomal position of the operon‐encoding *ypaA*. (b) Northern blot analysis of *ypaA* and the upstream FMN riboswitch. The transcripts were detected with specific probes in (1) wild type, (2) *Δrny*, (3) *Δrny* carrying an empty vector (Δ*rny*_pDL278), and (4) Δ*rny* complemented (Δ*rny*_pDL278::*rny*) strains

The *ypaA* gene encodes a riboflavin transporter. The gene is preceded by a typical flavin mononucleotide (FMN) riboswitch (Patenge et al., 2012) in its 5’ UTR and is followed by a putative phosphatase‐encoding gene (Spy49_0308) (Figure 7a). The FMN riboswitch functions as a riboflavin sensor that activates the *ypaA* gene expression in response to low levels of riboflavin. The *ypaA* gene was not induced under the condition used in this study since the culture medium was replete with riboflavin. Therefore, only the FMN riboswitch (0.2 kb transcript), but not the *ypaA* mRNA, was detected in the WT. The *ypaA* mRNA was detected in the ∆*rny* mutant as a 1.6 kb read through transcript (Figure 7b). Similar FMN riboswitch mRNA levels were observed in the WT and ∆*rny* mutant, which suggests that the expression of FMN riboswitch is unaffected by the RNase Y mutation. It also suggests that the FMN riboswitch is not a breakdown product of the *ypaA* read through, since the accumulation of *ypaA* read through in the ∆*rny* mutant does not lead to a reduction of the FMN riboswitch.

### 3.9 PrtF

**Figure 8:**
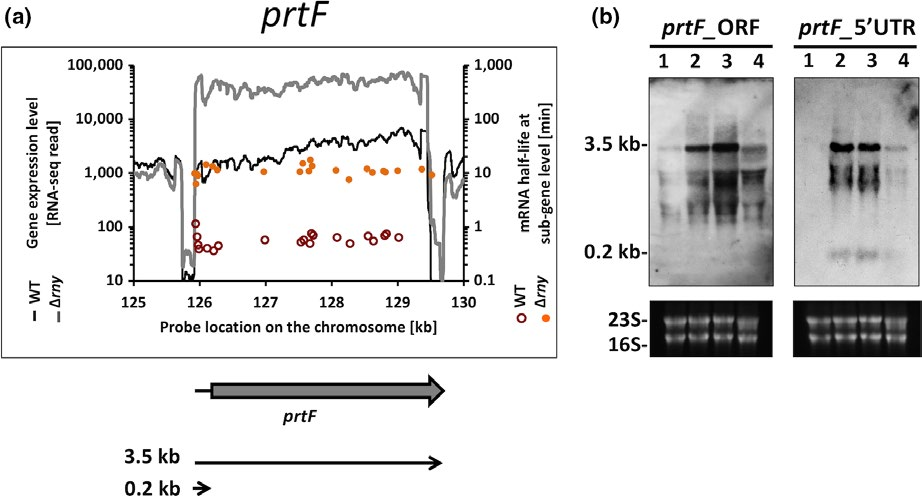
Role of RNaseY in transcript processing of *prtF*. (a) Analysis of gene expression levels and mRNA half‐lives of *prtF* in *Streptococcus pyogenes* NZ131 wild type and Δ*rny* mutant. Gene expression is presented as RNA‐seq reads and mRNA half‐life is presented as probe signal intensity relative to the chromosomal position of the *prtF* gene. (b) Northern blot analysis of *prtF* 5’ UTR and the *prtF* orf. The transcripts were detected with specific probes in (1) wild type, (2) *Δrny*, (3) *Δrny* carrying an empty vector (Δ*rny*_pDL278), and (4) Δ*rny* complemented (Δ*rny*_pDL278::*rny*) strains

The *prtF* gene encodes a fibronectin‐binding adhesin protein F that mediates binding of *S. pyogenes* to extracellular matrix and host cells (Fogg & Caparon, 1997) (Figure 8a). Northern blot analysis of the *prtF* mRNA with a CDS probe detected a full‐length mRNA (3.5 kb transcript) and multiple transcripts with reduced sizes in the WT. The same transcripts were observed in the ∆*rny* strain with stronger signal intensity, which suggested that these shorter transcripts were not processed mRNAs but were probably degradation intermediates. Similar expression patterns were observed with Northern blot analysis targeting the *prtF* 5’ UTR. An additional 0.2 kb transcript was found in the Δ*rny* mutant, which we presumed to be another degradation intermediate (Figure 8b).

### 3.10 Effects of altered mRNA levels on protein abundance

We questioned whether the alteration of mRNA expression in the Δ*rny* background correlated with differential protein production. Four proteins, SpeB, RopB, PrtF, and SpeG, were examined for their abundance in the WT, Δ*rny*, Δ*rny*_pDL278 and Δ*rny*_pDL278::*rny* strains. We were not able to examine the protein abundance of YpaA or FolC1 because of the lack of specific antibodies, and several attempts of protein tagging were unsuccessful.

**Figure 9:**
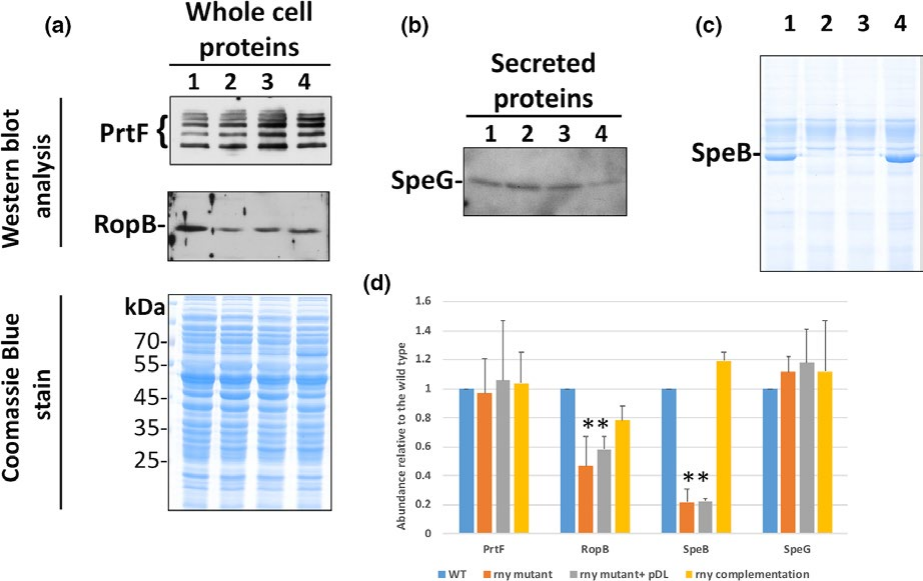
Detection of protein abundance of selected proteins. (a) Western blot analysis with RopB‐ and PrtF‐specific antibodies using whole‐cell preparations. The Coomassie Blue stained SDS‐gel page is shown as a loading control. (b) Western blot detection of secreted SpeG. (c) Coomassie Blue stained SDS‐page gel showing whole‐cell lysates from late exponential phase cells. SpeB is highly expressed in the late exponential growth phase and the protein is directly visible via SDS‐PAGE. (d) Quantification of band intensities using Image J. The abundance is presented relative to the wild type. For RopB, SpeG and Prtf *n* = 3; for SpeB *n* = 2. (* = significant difference; *p* ≤ 0.01, Student’s *t* test)

Western blots detected RopB, PrtF, and SpeG with specific antibodies (Figure 9a,b), while secreted SpeB was detected via SDS‐PAGE and Coomassie blue staining (Figure 9c). Similar amounts of the PrtF and SpeG proteins were quantified in all tested samples (Figure 9d), despite their significantly increased mRNA levels in the Δ*rny* mutant. Significantly reduced RopB protein production was observed in the Δ*rny* strain even though *ropB* mRNA levels were unaffected (Figure 9d). Significantly reduced SpeB production was observed in the Δ*rny* strain (Figure 9d), which was in accordance with the reduced *speB* mRNA level in that strain as reported before (Chen et al., 2013).

## 4 DISCUSSION

The aim of this study was the identification of new RNase Y mRNA targets in *S. pyogenes*. We previously demonstrated that RNase Y is the major endonuclease responsible for global mRNA turnover in *S. pyogenes* (Chen et al., 2013). Although not essential, RNase Y ensures rapid turnover of mRNA and a deletion of the *rny* gene increases the average mRNA half‐life from 1.24 min to 2.79 min (Chen et al., 2013). RNase Y activity also seems to be important during infection, since an RNase Y deletion mutant is highly attenuated in a murine subcutaneous infection model (Kang, Caparon, & Cho, 2010). During our initial studies, we observed that RNase Y is able to process primary transcripts. This was shown for *ropB* and *speB*, encoding the important transcriptional regulator that activates expression of streptococcus exotoxin B‐encoding gene (*speB*), a major *S. pyogenes* virulence factor (Chen et al., 2012, 2013).

Here, we combined RNA‐seq and tiling‐microarray analyses to search for additional RNase Y‐processed mRNAs in *S. pyogenes* on a genome‐wide scale. To do so, it was first necessary to predict the global operon structure in the cell. Overall 1699 genes were grouped into 865 operons, with 491 monocistrons. The median lengths of the 5’ UTRs and 3’ UTRs were predicted to be 43 nt and 33 nt, respectively (Figure 3a,b). Another *S. pyogenes* strain MGAS2221 (serotype M1) has been analyzed by RNA‐seq, though the complete transcriptome map is not publically available (McClure et al., 2013). Analysis of the MGAS2221 strain identified 412 polycistronic operons, which is comparable to our result. The average UTR lengths have not been reported for this strain. Our operon prediction also yielded similar results to those reported for related human pathogen *Streptococcus agalactiae*, which encodes 891 operons and 484 monocistrons (Rosinski‐Chupin et al., 2015). Moreover, the distribution of 5’ UTR lengths in strain NZ131 is proportional to the distribution reported for *S. agalactiae* and the swine pathogen *Streptococcus suis* (Rosinski‐Chupin et al., 2015; Wu et al., 2014). To further validate our results, we compared our operon predictions with a computational prediction using ProOpDB. Both our control experiments and the comparison to existing data from closely related streptococci support our operon prediction method.

After comparing the wild type and RNase Y mutant, transcript analysis narrowed our search for RNase Y‐processed mRNAs to 15 operons. Further experimental examination of four mRNAs was performed to verify if their transcripts serve as an RNase Y substrate. Three were either transcriptional read through products or regular unprocessed mRNAs and one (*folC1*) showed a truncated transcript independent of RNase Y. These observations do not support our original hypothesis that RNase Y‐mediated mRNA processing plays a significant role in global gene regulation, rather this enzyme seems to possess a more specific regulon.

The *folC1* mRNA was initially identified as RNase Y substrate based on the segmental stability pattern. FolC catalyzes the conversion of folates to polyglutamate (Bognar et al., 1987). Bacteria require folates for the biosynthesis of glycine, methionine, formylmethionine, thymidine, purines, and pantothenate (Bognar et al., 1987). Unlike other known processed mRNAs that are cleaved either in the UTRs or between ORFs, the *folC1* mRNA was processed in the middle of the protein‐coding region, but unlikely by RNase Y since the detected truncated transcript was equally abundant in the *rny* mutant and wild type. However, we observed an increase in mRNA stability for the *rny* mutant strain, but it is unclear if this is a direct effect. No functional enzyme activity was expected to result from the processed *folC1* mRNA, since only the 5’ half of the mRNA was expressed. Another copy of the *folC* gene, *folC2* (Spy49_0638), has been identified in the *S. pyogenes* genome. Sequence analysis showed 64% similarity between the two genes. Therefore, it is possible that *folC2* has redundant functions that can compensate under conditions when *folC1* is processed. However, the physiological implications of *folC1* processing and the exact mechanism how the truncated transcript is generated still requires further investigation.

While the number of RNase Y‐processed transcripts is apparently low, it affects important virulence traits of *S. pyogenes* (Chen et al., 2013; Kang et al., 2010) and Figures S2 and S3. The importance of RNase Y targets *ropB* and *speB* for *S. pyogenes* virulence is well established (Carroll & Musser, 2011). Interestingly, a recent study of RNase Y‐dependent mRNA decay and processing in pathogenic *Clostridium perfringens* demonstrated a similar effect on global mRNA stability, but similarly identified only two transcripts stabilized by RNase Y processing (Obana, Nakamura, & Nomura, 2017). The first, *colA*, requires RNase Y‐dependent processing to allow access to its ribosome‐binding site and subsequent translation into the major toxin collagenase. The second, *pilA2*, is stabilized after processing and results in the production of a major pilin component of the type IV pili important for biofilm formation and host cell adherence (Obana et al., 2017). A difference in the role of RNase Y was observed in *S. aureus*:* S. aureus* lacking RNase Y only showed stabilization of some operons, but is not responsible for global mRNA turnover suggesting that RNase Y has a more specialized function in this organism (Marincola et al., 2012). Initially, the transcript of the *sae* operon, which encodes a major virulence regulator, was identified as RNase Y processed (Marincola et al., 2012) and further examination nicely showed that a secondary structure downstream of the cleavage site is important to guide RNase Y for mRNA cleavage, but the actual cleavage site is not important for the cleavage process (Marincola & Wolz, 2017) A more detailed study showed that 248 orfs are strongly affected by RNase Y. Interestingly, the authors also demonstrated that subcellular localization of RNase Y in *S. aureus* via its membrane anchor restricts its target choice (Khemici, Prados, Linder, & Redder, 2015).

Although our approach identified RNase Y‐processed mRNAs, it has several limitations. First, segmental stability is not a unique character of processed mRNAs, but can also be observed in operons containing multiple promoters as well as through transcriptional read through. To reduce these false positives, we subtracted “processed” mRNA candidates of the RNase Y mutant from those of the WT so that the remaining mRNAs were most likely processed by RNase Y. This approach reduced the number of mRNA candidates from 80 to 26, but apparently did not completely prevent the detection of false positives. An alternative approach is to separate primary mRNAs (which are 5’ triphosphorylated) from processed mRNAs (which are 5’ monophosphorylated) and then analyze them individually (Jager, Forstner, Sharma, Santangelo, & Reeve, 2014). However, this approach cannot differentiate between processed mRNA and degraded fragments, which again would lead to false positives. The combination of the two approaches may yield more precise predictions. Second, the precise determination of transcriptional start sites (TSSs) at a single‐nucleotide resolution requires the application of differential RNA sequencing (dRNA‐seq) (Borries, Vogel, & Sharma, 2012), which was not available for this study. Alternatively, we used whole‐transcript RNA‐seq datasets to estimate operon boundaries. The estimation is not as accurate as that of dRNA‐seq because the reads are relatively depleted toward the 5’ and 3’ ends (Wang, Gerstein, & Snyder, 2009). However, for the purpose of our study, it was also unnecessary to determine the exact TSS of each operon.

Furthermore, our results clearly indicate the involvement of RNase Y in mRNA processing and read through degradation. However, direct evidence showing that RNase Y is responsible for the cleavage is absent. Recombinant *B. subtilis* RNase Y has been successfully purified from *E. coli* and displayed the ability to degrade SAM‐dependent riboswitches in vitro (Shahbabian, Jamalli, Zig, & Putzer, 2009). We were able to purify recombinant *S. pyogenes* RNase Y from *E. coli*, but the recombinant protein did not display any endonucleolytic activity in vitro (data not shown).

Surprisingly, we did not find an obvious correlation between the mRNA level and protein abundance of the genes examined in this study. The *speG* and *prtF* mRNAs, which were strongly stabilized and overexpressed in the Δ*rny* mutant, yielded similar amounts of protein to the WT. Conversely, the *ropB* mRNA was processed in the WT, but not in the Δ*rny* mutant. In this case, a lack of *ropB* processing in the Δ*rny* mutant led to reduced RopB protein abundance. The poor correlation between mRNA and protein levels has also been observed in other bacterial species (Dressaire, Laurent, Loubiere, Besse, & Cocaign‐Bousquet, 2010; Lu, Vogel, Wang, Yao, & Marcotte, 2007; Picard et al., 2012), which implies the involvement of translational regulation. Picard et al. reported a strong inverse correlation between mRNA stability and ribosome occupancy (the fraction of mRNA engaged in translation) and ribosome density in *Lactococcus lactis* (Picard et al., 2012). Both ribosome occupancy and ribosome density are major determinants of final protein expression levels. The authors reasoned that mRNAs with less stable secondary structures in the 5’ UTR would be more rapidly degraded on one hand but could be better translated with higher level of ribosome occupancy on the other hand. Consequently, mRNAs with higher turnover rates also have higher protein production rates. Thus, it is possible that the *speG* read through transcript is poorly translated because of strong secondary structures in their 5’ regions (Figure S4). Similarly, the unprocessed *ropB* mRNA created as a result of an RNase Y mutation is presumably translated at a lower efficiency than the processed mRNA because of two step‐loop structures in the 5’ UTR (Neely, Lyon, Runft, & Caparon, 2003).

## CONFLICT OF INTEREST

The authors declare no conflict of interest.

## References

[mbo3671-bib-0001] Ajdic, D. , & Pham, V. T. (2007). Global transcriptional analysis of *Streptococcus mutans* sugar transporters using microarrays. Journal of Bacteriology, 189, 5049–5059. 10.1128/JB.00338-07 17496079 PMC1951856

[mbo3671-bib-0002] Bechhofer, D. H. (2009). Messenger RNA decay and maturation in *Bacillus subtilis* . Prog Mol Biol Transl Sci, 85, 231–273. 10.1016/S0079-6603(08)00806-4 19215774

[mbo3671-bib-0003] Bognar, A. L. , Osborne, C. , & Shane, B. (1987). Primary structure of the *Escherichia coli folC* gene and its folylpolyglutamate synthetase‐dihydrofolate synthetase product and regulation of expression by an upstream gene. Journal of Biological Chemistry, 262, 12337–12343. 3040739

[mbo3671-bib-0004] Borries, A. , Vogel, J. , & Sharma, C. M. (2012). Differential RNA sequencing (dRNA‐Seq): Deep‐sequencing‐based analysis of primary transcriptomes In Harbers M. & Kahl G. (Eds.), Tag‐based next generation sequencing. (pp. 109‐122) Weinheim: Wiley‐VCH Verlag GmbH & Co. KGaA.

[mbo3671-bib-0005] Carroll, R. K. , & Musser, J. M. (2011). From transcription to activation: How group A streptococcus, the flesh‐eating pathogen, regulates SpeB cysteine protease production. Molecular Microbiology, 81, 588–601. 10.1111/j.1365-2958.2011.07709.x 21707787

[mbo3671-bib-0006] Chen, Z. , Itzek, A. , Malke, H. , Ferretti, J. J. , & Kreth, J. (2012). Dynamics of *speB* mRNA transcripts in *Streptococcus pyogenes* . Journal of Bacteriology, 194, 1417–1426. 10.1128/JB.06612-11 22267517 PMC3294869

[mbo3671-bib-0007] Chen, Z. , Itzek, A. , Malke, H. , Ferretti, J. J. , & Kreth, J. (2013). Multiple roles of RNase Y in *Streptococcus pyogenes* mRNA processing and degradation. Journal of Bacteriology, 195, 2585–2594. 10.1128/JB.00097-13 23543715 PMC3676074

[mbo3671-bib-0008] Condon, C. (2007). Maturation and degradation of RNA in bacteria. Current Opinion in Microbiology, 10, 271–278. 10.1016/j.mib.2007.05.008 17560162

[mbo3671-bib-0009] Condon, C. , & Bechhofer, D. H. (2011). Regulated RNA stability in the Gram positives. Current Opinion in Microbiology, 14, 148–154. 10.1016/j.mib.2011.01.010 21334965 PMC3078962

[mbo3671-bib-0010] Deltcheva, E. , Chylinski, K. , Sharma, C. M. , Gonzales, K. , Chao, Y. , Pirzada, Z. A. , … Charpentier, E. (2011). CRISPR RNA maturation by trans‐encoded small RNA and host factor RNase III. Nature, 471, 602–607. 10.1038/nature09886 21455174 PMC3070239

[mbo3671-bib-0011] Deutscher, M. P. (2006). Degradation of RNA in bacteria: Comparison of mRNA and stable RNA. Nucleic Acids Research, 34, 659–666. 10.1093/nar/gkj472 16452296 PMC1360286

[mbo3671-bib-0012] Dressaire, C. , Laurent, B. , Loubiere, P. , Besse, P. , & Cocaign‐Bousquet, M. (2010). Linear covariance models to examine the determinants of protein levels in *Lactococcus lactis* . Molecular BioSystems, 6, 1255–1264. 10.1039/c001702g 20448864

[mbo3671-bib-0013] Fogg, G. C. , & Caparon, M. G. (1997). Constitutive expression of fibronectin binding in *Streptococcus pyogenes* as a result of anaerobic activation of *rofA* . Journal of Bacteriology, 179, 6172–6180. 10.1128/jb.179.19.6172-6180.1997 9324268 PMC179524

[mbo3671-bib-0014] Herskovits, A. A. , Bochkareva, E. S. , & Bibi, E. (2000). New prospects in studying the bacterial signal recognition particle pathway. Molecular Microbiology, 38, 927–939. 11123669 10.1046/j.1365-2958.2000.02198.x

[mbo3671-bib-0015] Jager, D. , Forstner, K. U. , Sharma, C. M. , Santangelo, T. J. , & Reeve, J. N. (2014). Primary transcriptome map of the hyperthermophilic archaeon *Thermococcus kodakarensis* . BMC Genomics, 15, 684 10.1186/1471-2164-15-684 25127548 PMC4247193

[mbo3671-bib-0016] Kang, S. O. , Caparon, M. G. , & Cho, K. H. (2010). Virulence gene regulation by CvfA, a putative RNase: The CvfA‐enolase complex in *Streptococcus pyogenes* links nutritional stress, growth‐phase control, and virulence gene expression. Infection and Immunity, 78, 2754–2767. 10.1128/IAI.01370-09 20385762 PMC2876558

[mbo3671-bib-0017] Khemici, V. , Prados, J. , Linder, P. , & Redder, P. (2015). Decay‐initiating endoribonucleolytic cleavage by RNase Y is kept under tight control via sequence preference and sub‐cellular localisation. PLoS Genetics, 11, e1005577 10.1371/journal.pgen.1005577 26473962 PMC4608709

[mbo3671-bib-0018] Kreth, J. , Chen, Z. , Ferretti, J. , & Malke, H. (2011). Counteractive balancing of transcriptome expression involving CodY and CovRS in *Streptococcus pyogenes* . Journal of Bacteriology, 193, 4153–4165. 10.1128/JB.00061-11 21705595 PMC3147680

[mbo3671-bib-0019] Langmead, B. , & Salzberg, S. L. (2012). Fast gapped‐read alignment with Bowtie 2. Nature Methods, 9, 357–359. 10.1038/nmeth.1923 22388286 PMC3322381

[mbo3671-bib-0020] Li, H. , Handsaker, B. , Wysoker, A. , Fennell, T. , Ruan, J. , Homer, N. , … Durbin, R. (2009). The sequence alignment/map format and SAMtools. Bioinformatics, 25, 2078–2079. 10.1093/bioinformatics/btp352 19505943 PMC2723002

[mbo3671-bib-0021] Lodish, H. , Berk, A. , Zipursky, S. L. , Matsudaira, P. , Baltimore, D. , & Darnell, J. (2000). Molecular cell biology. New York: MacMillan.

[mbo3671-bib-0022] Lu, P. , Vogel, C. , Wang, R. , Yao, X. , & Marcotte, E. M. (2007). Absolute protein expression profiling estimates the relative contributions of transcriptional and translational regulation. Nature Biotechnology, 25, 117–124. 10.1038/nbt1270 17187058

[mbo3671-bib-0023] Ludwig, H. , Homuth, G. , Schmalisch, M. , Dyka, F. M. , Hecker, M. , & Stulke, J. (2001). Transcription of glycolytic genes and operons in *Bacillus subtilis*: Evidence for the presence of multiple levels of control of the *gapA* operon. Molecular Microbiology, 41, 409–422. 10.1046/j.1365-2958.2001.02523.x 11489127

[mbo3671-bib-0024] Ludwig, H. , Rebhan, N. , Blencke, H. M. , Merzbacher, M. , & Stulke, J. (2002). Control of the glycolytic *gapA* operon by the catabolite control protein A in *Bacillus subtilis*: A novel mechanism of CcpA‐mediated regulation. Molecular Microbiology, 45, 543–553. 10.1046/j.1365-2958.2002.03034.x 12123463

[mbo3671-bib-0025] Lyon, W. R. , Gibson, C. M. , & Caparon, M. G. (1998). A role for trigger factor and an *rgg*‐like regulator in the transcription, secretion and processing of the cysteine proteinase of *Streptococcus pyogenes* . EMBO Journal, 17, 6263–6275. 10.1093/emboj/17.21.6263 9799235 PMC1170952

[mbo3671-bib-0026] Mader, U. , Hennig, S. , Hecker, M. , & Homuth, G. (2004). Transcriptional organization and posttranscriptional regulation of the *Bacillus subtilis* branched‐chain amino acid biosynthesis genes. Journal of Bacteriology, 186, 2240–2252. 10.1128/JB.186.8.2240-2252.2004 15060025 PMC412147

[mbo3671-bib-0027] Marincola, G. , Schafer, T. , Behler, J. , Bernhardt, J. , Ohlsen, K. , Goerke, C. , & Wolz, C. (2012). RNase Y of *Staphylococcus aureus* and its role in the activation of virulence genes. Molecular Microbiology, 85, 817–832. 10.1111/j.1365-2958.2012.08144.x 22780584

[mbo3671-bib-0028] Marincola, G. , & Wolz, C. (2017). Downstream element determines RNase Y cleavage of the *saePQRS* operon in *Staphylococcus aureus* . Nucleic Acids Research, 45, 5980–5994. 10.1093/nar/gkx296 28453818 PMC5449607

[mbo3671-bib-0029] McClure, R. , Balasubramanian, D. , Sun, Y. , Bobrovskyy, M. , Sumby, P. , Genco, C. A. , … Tjaden, B. (2013). Computational analysis of bacterial RNA‐Seq data. Nucleic Acids Research, 41, e140 10.1093/nar/gkt444 23716638 PMC3737546

[mbo3671-bib-0030] McShan, W. M. , Ferretti, J. J. , Karasawa, T. , Suvorov, A. N. , Lin, S. , Qin, B. , … Savic, D. J. (2008). Genome sequence of a nephritogenic and highly transformable M49 strain of *Streptococcus pyogenes* . Journal of Bacteriology, 190, 7773–7785. 10.1128/JB.00672-08 18820018 PMC2583620

[mbo3671-bib-0031] Meinken, C. , Blencke, H. M. , Ludwig, H. , & Stulke, J. (2003). Expression of the glycolytic *gapA* operon in *Bacillus subtilis*: Differential syntheses of proteins encoded by the operon. Microbiology, 149, 751–761. 10.1099/mic.0.26078-0 12634343

[mbo3671-bib-0032] Nazar, R. N. (2004). Ribosomal RNA processing and ribosome biogenesis in eukaryotes. IUBMB Life, 56, 457–465. 10.1080/15216540400010867 15545225

[mbo3671-bib-0033] Neely, M. N. , Lyon, W. R. , Runft, D. L. , & Caparon, M. (2003). Role of RopB in growth phase expression of the SpeB cysteine protease of *Streptococcus pyogenes* . Journal of Bacteriology, 185, 5166–5174. 10.1128/JB.185.17.5166-5174.2003 12923089 PMC181010

[mbo3671-bib-0034] Nelson, D. , Schuch, R. , Chahales, P. , Zhu, S. , & Fischetti, V. A. (2006). PlyC: A multimeric bacteriophage lysin. Proceedings of the National Academy of Sciences of the United States of America, 103, 10765–10770. 10.1073/pnas.0604521103 16818874 PMC1487170

[mbo3671-bib-0035] Obana, N. , Nakamura, K. , & Nomura, N. (2017). Role of RNase Y in *Clostridium perfringens* mRNA decay and processing. Journal of Bacteriology, 199, pii: e00703–e00716. 27821608 10.1128/JB.00703-16 PMC5198488

[mbo3671-bib-0036] Patenge, N. , Billion, A. , Raasch, P. , Normann, J. , Wisniewska‐Kucper, A. , Retey, J. , … Kreikemeyer, B. (2012). Identification of novel growth phase‐ and media‐dependent small non‐coding RNAs in *Streptococcus pyogenes* M49 using intergenic tiling arrays. BMC Genomics, 13, 550 10.1186/1471-2164-13-550 23062031 PMC3542284

[mbo3671-bib-0037] Picard, F. , Milhem, H. , Loubiere, P. , Laurent, B. , Cocaign‐Bousquet, M. , & Girbal, L. (2012). Bacterial translational regulations: High diversity between all mRNAs and major role in gene expression. BMC Genomics, 13, 528 10.1186/1471-2164-13-528 23036066 PMC3543184

[mbo3671-bib-0038] Quinlan, A. R. , & Hall, I. M. (2010). BEDTools: A flexible suite of utilities for comparing genomic features. Bioinformatics, 26, 841–842. 10.1093/bioinformatics/btq033 20110278 PMC2832824

[mbo3671-bib-0039] Remmele, C. W. , Xian, Y. , Albrecht, M. , Faulstich, M. , Fraunholz, M. , Heinrichs, E. , … Rudel, T. (2014). Transcriptional landscape and essential genes of *Neisseria gonorrhoeae* . Nucleic Acids Research, 42, 10579–10595. 10.1093/nar/gku762 25143534 PMC4176332

[mbo3671-bib-0040] Rosinski‐Chupin, I. , Sauvage, E. , Sismeiro, O. , Villain, A. , da Cunha, V. , Caliot, M. E. , … Glaser, P. (2015). Single nucleotide resolution RNA‐seq uncovers new regulatory mechanisms in the opportunistic pathogen *Streptococcus agalactiae* . BMC Genomics, 16, 419 10.1186/s12864-015-1583-4 26024923 PMC4448216

[mbo3671-bib-0041] Shahbabian, K. , Jamalli, A. , Zig, L. , & Putzer, H. (2009). RNase Y, a novel endoribonuclease, initiates riboswitch turnover in *Bacillus subtilis* . EMBO Journal, 28, 3523–3533. 10.1038/emboj.2009.283 19779461 PMC2782095

[mbo3671-bib-0042] Steer, A. C. , Lamagni, T. , Curtis, N. , & Carapetis, J. R. (2012). Invasive group a streptococcal disease: Epidemiology, pathogenesis and management. Drugs, 72, 1213–1227. 10.2165/11634180-000000000-00000 22686614 PMC7100837

[mbo3671-bib-0043] Taboada, B. , Ciria, R. , Martinez‐Guerrero, C. E. , & Merino, E. (2012). ProOpDB: Prokaryotic Operon DataBase. Nucleic Acids Research, 40, D627–D631. 10.1093/nar/gkr1020 22096236 PMC3245079

[mbo3671-bib-0044] Wang, Z. , Gerstein, M. , & Snyder, M. (2009). RNA‐Seq: A revolutionary tool for transcriptomics. Nature Reviews Genetics, 10, 57–63. 10.1038/nrg2484 PMC2949280 19015660

[mbo3671-bib-0045] Wu, Z. , Wu, C. , Shao, J. , Zhu, Z. , Wang, W. , Zhang, W. , … Lu, C. (2014). The *Streptococcus suis* transcriptional landscape reveals adaptation mechanisms in pig blood and cerebrospinal fluid. RNA, 20, 882–898. 10.1261/rna.041822.113 24759092 PMC4024642

