# Supplementary material for: Genome‐wide screening of potential RNase Y‐processed mRNAs in the M49 serotype Streptococcus pyogenes NZ131
Source: Microbiologyopen. 2018 Jun 13;8(4):e00671. doi: 10.1002/mbo3.671 (PMC6460267; doi:10.1002/mbo3.671)
Supplement: Supplementary file 1 [file MBO3-8-e00671-s001.pdf]

supplemental materials

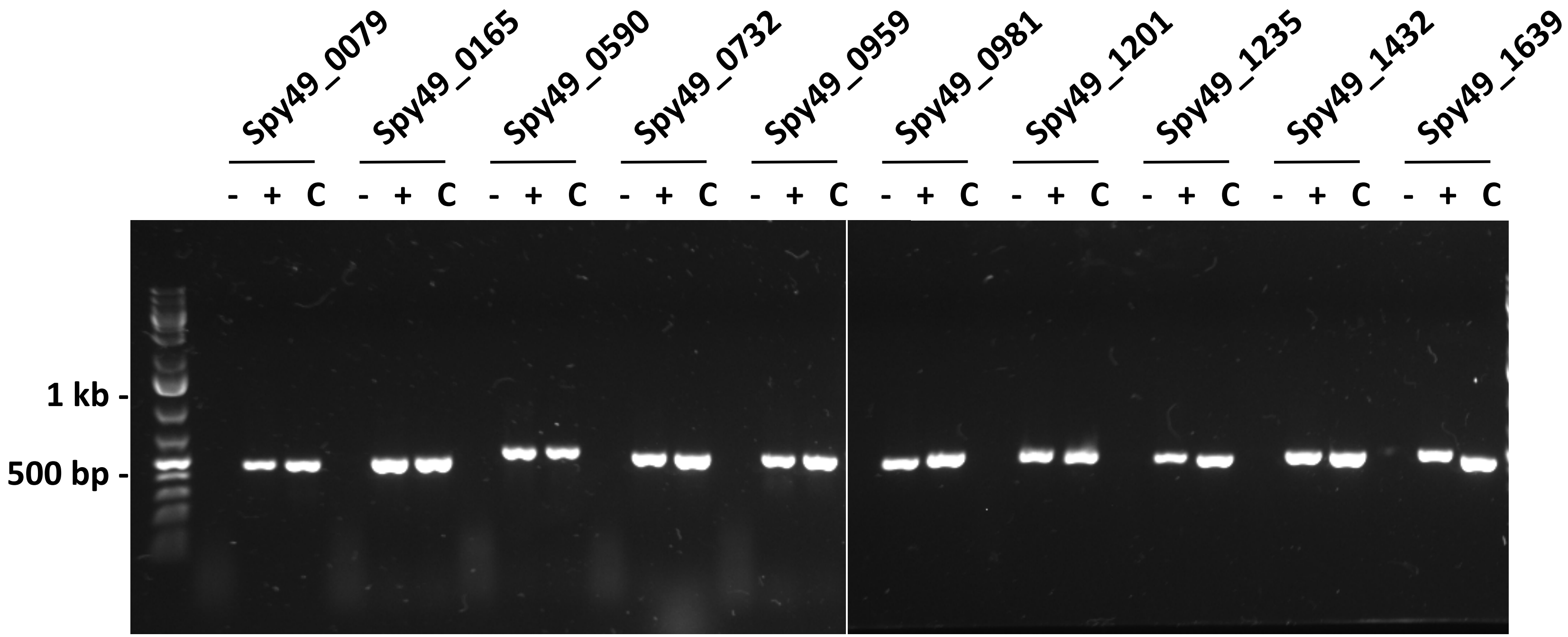

Fig. S1: Reverse transcription PCR (RT-PCR) analysis of the co-transcription of two adjacent genes on the chromosome. Ten gene pairs were selected. The locus of the first gene in each gene pair was indicated. PCR primers were designed so that the forward primer hybridizes to the first gene and the reverse primer hybridizes to the second gene for each gene pair. Total RNA and chromosomal DNA were used as negative (-) and positive (+) controls. cDNA (C) obtained from reverse transcription of total RNA was used as test sample.

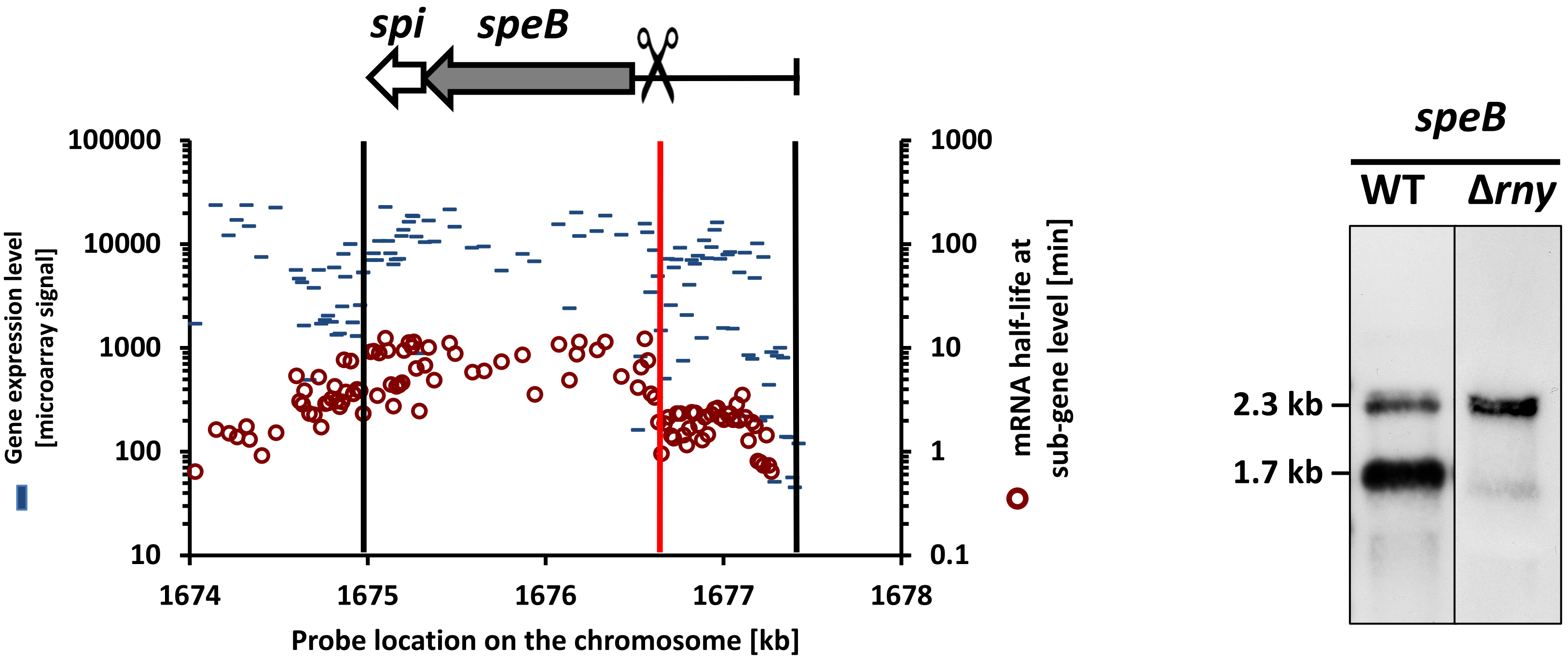

Fig. S2: Analysis of gene expression levels and mRNA half-lives of *speB* in *S. pyogenes* NZ131 wild type and *Δrny* mutant. Gene expression is presented as RNA-seq reads and mRNA half-life is presented as probe signal intensity relative to the chromosomal position of the *speB* encoding operon. Northern blot analysis of *speB.* The transcripts were detected with specific probes in (1) wild type, (2) *Δrny* strains.

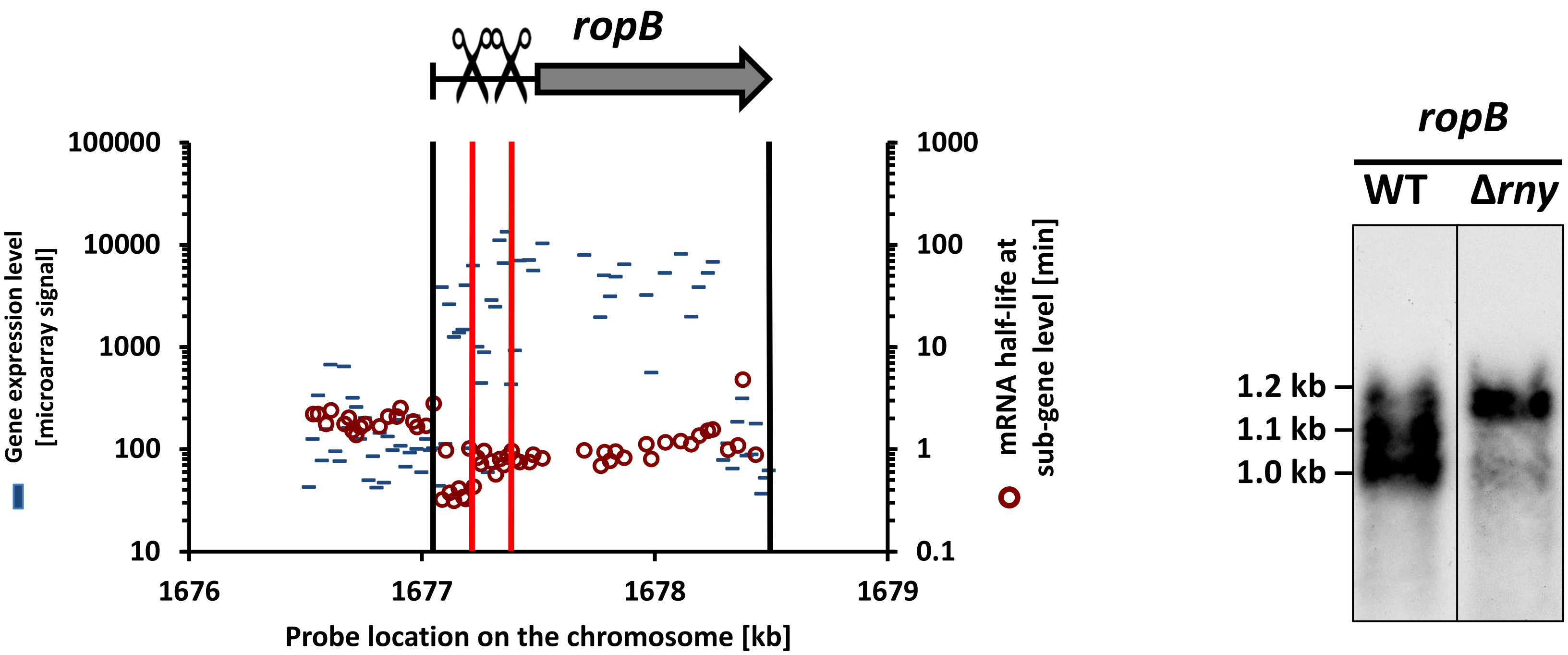

Fig. S3: Analysis of gene expression levels and mRNA half-lives of *ropB* in *S. pyogenes* NZ131 wild type and *Δrny* mutant. Gene expression is presented as RNA-seq reads and mRNA half-life is presented as probe signal intensity relative to the chromosomal position of the *ropB* encoding operon. Northern blot analysis of *ropB* . The transcripts were detected with specific probes in (1) wild type, (2) *Δrny* strains.

Output of sir_graph (©)
mfold_util 4.7

Created Fri May 19 18:42:02 2017

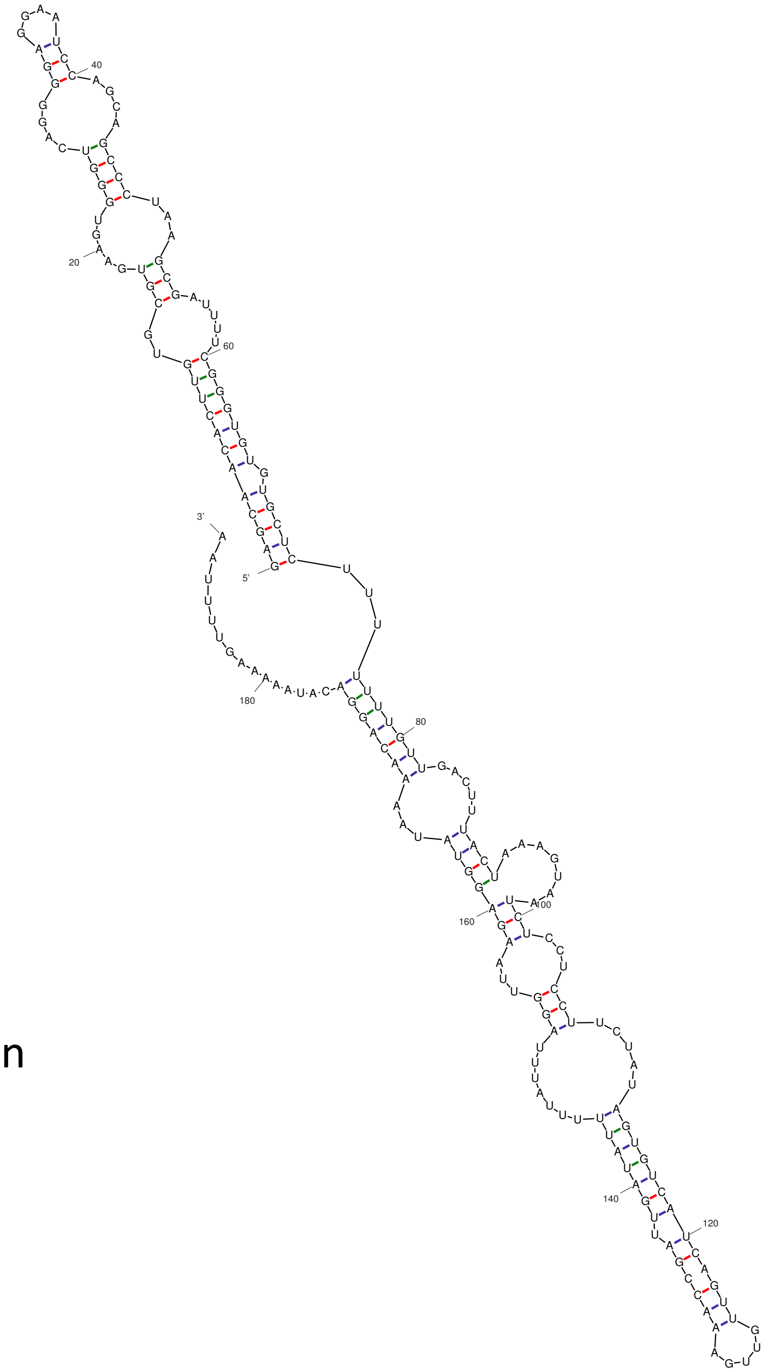

Fig. S4: Mfold analysis of the *speG* 5’ region

dG = -44.20 [Initially -44.20] 17May19-18-42-00

Table S1. Statistical analysis of RNA-seq reads mapped to the *S. pyogenes* NZ131 genome.

	WT1		WT2		Δrny1		Δrny2	
	Count	Percentage of reads	Count	Percentage of reads	Count	Percentage of reads	Count	Percentage of reads
**Mapped reads**	110,875,130	99.47%	110,557,162	99.12%	118,067,816	99.11%	121,808,330	99.06%
**Not mapped reads**	593,828	0.53%	979,588	0.88%	1,061,748	0.89%	1,150,506	0.94%
**Reads in pairs**	101,959,594	91.47%	106,229,606	95.24%	111,198,824	93.34%	113,291,016	92.14%
**Broken paired reads**	8,915,536	8.00%	4,327,556	3.88%	6,868,992	5.77%	8,517,314	6.93%
**Total reads**	111,468,958	100.00%	111,536,750	100.00%	119,129,564	100.00%	122,958,836	100.00%
