# Supplementary material for: Genome‐wide screening of potential RNase Y‐processed mRNAs in the M49 serotype Streptococcus pyogenes NZ131
Source: Microbiologyopen. 2018 Jun 13;8(4):e00671. doi: 10.1002/mbo3.671 (PMC6460267; doi:10.1002/mbo3.671)
Supplement: Supplementary file 2 [file MBO3-8-e00671-s002.pdf]

Operon	Orientation	Genes	LeftBound	LeftBound Confidence
operon_1	+	Spy49_0001	231	Low
operon_2	+	Spy49_0002	1665	Low
operon_3	+	Spy49_0003	2937	High
operon_4	+	Spy49_0004	3454	High
operon_5	+	Spy49_0005, Spy49_0006	4630	Low
operon_6	+	Spy49_0008, Spy49_0009	8821	Low
operon_7	+	Spy49_0010, Spy49_0011, Spy49_0012, Spy49_0013	9600	Low
operon_8	+	Spy49_0014	14802	Low
operon_9	+	Spy49_rRNA16s1	17126	High
operon_10	+	Spy49_rRNA23s1	18831	High
operon_11	+	Spy49_rRNA5s5	22004	High
operon_12	+	Spy49_tRNAasp1, Spy49_tRNAasp2	22151	High
operon_13	+	Spy49_tRNAlys1	22331	High
operon_14	+	Spy49_tRNAgly1, Spy49_tRNAleu4, Spy49_tRNAarg6	22517	Low
operon_15	+	Spy49_rRNA16s5	23076	High
operon_16	+	Spy49_tRNAala3, Spy49_tRNAala4	24664	High
operon_17	+	Spy49_rRNA23s2	25183	High
operon_18	+	Spy49_rRNA5s3	28004	High
operon_19	+	Spy49_tRNAasp3	28150	High
operon_20	+	Spy49_tRNAlys2, Spy49_tRNAleu1, Spy49_tRNAleu6, Spy49_tRNAthr1, Spy49_tRNAthr2, Spy49_tRNAgly2, Spy49_tRNAleu2, Spy49_tRNAarg1, Spy49_tRNApro1, Spy49_tRNApro2, Spy49_tRNAmet1, Spy49_tRNAmet2, Spy49_tRNApseudo1, Spy49_tRNAmet3, Spy49_tRNAphe2, Spy49_tRNAgly3	28332	High
operon_21	+	Spy49_tRNAile2, Spy49_tRNApseudo2	29498	High
operon_22	+	Spy49_0015	31138	High
operon_23	+	Spy49_0016	32595	High
operon_24	+	Spy49_0017	33714	High
operon_25	+	Spy49_0018, Spy49_0019	34573	Low
operon_26	+	Spy49_0020, Spy49_0021	35917	High
operon_27	+	Spy49_0022, Spy49_0023	40688	Low
operon_28	+	Spy49_0024	43393	Low
operon_29	+	Spy49_0025	44123	Low
operon_30	-	Spy49_0026c	45760	Low

operon_31	+	Spy49_0027	47026	Low
operon_32	+	Spy49_0028, Spy49_0029	48542	Low
operon_33	+	Spy49_0030	50229	High
operon_34	+	Spy49_0031	51962	Low
operon_35	+	Spy49_0032	53454	High
operon_36	+	Spy49_0033	54615	High
operon_37	+	Spy49_0035, Spy49_0036, Spy49_0037	55746	High
operon_38	+	Spy49_0038	58651	High
operon_39	+	Spy49_0039	61545	High
operon_40	+	Spy49_0040	62682	Low
operon_41	+	Spy49_0044	64350	High
operon_42	+	Spy49_0045, Spy49_0046, Spy49_0048, Spy49_0049	64913	Low
operon_43	+	Spy49_0050, Spy49_0052, Spy49_0053, Spy49_0054, Spy49_0055, Spy49_0056, Spy49_0057, Spy49_0058, Spy49_0059, Spy49_0060	67528	Low
operon_44	+	Spy49_0061	71440	High
operon_45	+	Spy49_0062	72208	High
operon_46	+	Spy49_0064, Spy49_0065, Spy49_0066	72705	Low
operon_47	+	Spy49_0067, Spy49_0068	73931	Low
operon_48	+	Spy49_0069	75850	High
operon_49	+	Spy49_0070, Spy49_0071, Spy49_0072, Spy49_0073, Spy49_0074, Spy49_0075	76644	High
operon_50	+	Spy49_rRNA16s3	79405	High
operon_51	+	Spy49_tRNAala1	81650	High
operon_52	+	Spy49_rRNA23s3	81727	High
operon_53	+	Spy49_rRNA5s2	85001	High
operon_54	+	Spy49_tRNAval4	85146	High
operon_55	+	Spy49_tRNAgly4	85229	High
operon_56	+	Spy49_tRNAglu2, Spy49_tRNApseudo3, Spy49_tRNAmet4, Spy49_tRNAphe1, Spy49_tRNAtyr1, Spy49_tRNAtyr2, Spy49_tRNAtrp1, Spy49_tRNAhis1, Spy49_tRNAgln2	85314	High
operon_57	+	Spy49_tRNAleu5	86131	Low
operon_58	+	Spy49_0078, Spy49_0079	86359	High
operon_59	+	Spy49_0080, Spy49_0081, Spy49_0082	87937	High
operon_60	-	Spy49_0083c	90016	High
operon_61	-	Spy49_0084	90428	High
operon_62	+	Spy49_0086	91788	High
operon_63	+	Spy49_0087	94232	Low

operon_64	+	Spy49_0088	97989	Low
operon_65	+	Spy49_0089	101793	High
operon_66	+	Spy49_0090	102215	Low
operon_67	+	Spy49_0091, Spy49_0092, Spy49_0093, Spy49_0094, Spy49_0095, Spy49_0096	103258	Low
operon_68	+	Spy49_0097	105987	High
operon_69	+	Spy49_0098	106970	Low
operon_70	+	Spy49_0099	108288	Low
operon_71	-	Spy49_0100, Spy49_0101	108733	High
operon_72	+	Spy49_0103, Spy49_0104, Spy49_0105	112445	High
operon_73	+	Spy49_0107	113830	High
operon_74	-	Spy49_0108c, Spy49_0109c, Spy49_0110c	114406	High
operon_75	-	Spy49_0111	117057	Low
operon_76	+	Spy49_0112, Spy49_0113, Spy49_0114, Spy49_0116, Spy49_0117	118881	High
operon_77	-	Spy49_0118	124314	Low
operon_78	+	Spy49_0119	125913	High
operon_79	-	Spy49_0120c	129680	High
operon_80	+	Spy49_0121	130516	High
operon_81	-	Spy49_0122c	132194	Low
operon_82	+	Spy49_0123, Spy49_0124, Spy49_0125	133242	High
operon_83	-	Spy49_0126c	135865	High
operon_84	+	Spy49_0127, Spy49_0128	136723	High
operon_85	+	Spy49_0129, Spy49_0130, Spy49_0131, Spy49_0132, Spy49_0133, Spy49_0134	138422	High
operon_86	+	Spy49_0135, Spy49_0136, Spy49_0137	143495	Low
operon_87	-	Spy49_0138c, Spy49_0139c	147541	High
operon_88	+	Spy49_0141	150010	High
operon_89	+	Spy49_0142	151624	Low
operon_90	+	Spy49_0143	152946	High
operon_91	+	Spy49_0144, Spy49_0145, Spy49_0146	153904	High
operon_92	+	Spy49_0147	157722	High
operon_93	+	Spy49_0148	158495	High
operon_94	-	Spy49_0149c	158948	High
operon_95	-	Spy49_0150c	159315	Low
operon_96	+	Spy49_0151	159580	High
operon_97	+	Spy49_0152	161484	High
operon_98	+	Spy49_0153, Spy49_0154	164280	High
operon_99	+	Spy49_0155, Spy49_0156, Spy49_0157, Spy49_0158	166162	Low
operon_100	+	Spy49_0159	169332	High
operon_101	+	Spy49_0160	171249	High

operon_102	+	Spy49_0161, Spy49_0162	172700	High
operon_103	+	Spy49_0163	175910	High
operon_104	+	Spy49_0164, Spy49_0165	178643	Low
operon_105	+	Spy49_0166	179784	Low
operon_106	+	Spy49_0167, Spy49_0168	180272	Low
operon_107	+	Spy49_0169	182063	High
operon_108	-	Spy49_0170c	182507	Low
operon_109	-	Spy49_0173c	183106	Low
operon_110	+	Spy49_0176	185222	High
operon_111	-	Spy49_0178c	186871	High
operon_112	-	Spy49_0180c	187889	High
operon_113	+	Spy49_0181	188911	High
operon_114	+	Spy49_0182, Spy49_0183	190367	High
operon_115	+	Spy49_0184, Spy49_0185	191347	High
operon_116	-	Spy49_0186c	193254	High
operon_117	+	Spy49_SRP	194719	High
operon_118	+	Spy49_0187	194849	Low
operon_119	+	Spy49_0188	196042	High
operon_120	-	Spy49_0189c	197573	High
operon_121	+	Spy49_0190	199641	High
operon_122	-	Spy49_0191c, Spy49_0192c	200719	High
operon_123	+	Spy49_0193, Spy49_0194, Spy49_0195	202954	High
operon_124	+	Spy49_0198	206929	Low
operon_125	+	Spy49_0199, Spy49_0200	207882	High
operon_126	+	Spy49_0201	209891	Low
operon_127	+	Spy49_0202	210588	High
operon_128	+	Spy49_0203	211493	High
operon_129	+	Spy49_0204, Spy49_0205, Spy49_0206	213329	High
operon_130	+	Spy49_0207	217242	High
operon_131	+	Spy49_0208, Spy49_0209	217608	Low
operon_132	+	Spy49_0210	219589	High
operon_133	+	Spy49_0211, Spy49_0212	220018	High
operon_134	+	Spy49_0213, Spy49_0214	222118	Low
operon_135	+	Spy49_0215, Spy49_0216, Spy49_0217	224072	Low
operon_136	-	Spy49_0218c	226726	Low
operon_137	+	Spy49_0219, Spy49_0220	227737	Low
operon_138	+	Spy49_0221	229306	Low
operon_139	+	Spy49_0223, Spy49_0224, Spy49_0225, Spy49_0226, Spy49_0227	230643	High
operon_140	+	Spy49_0228	235277	High
operon_141	+	Spy49_0229	236133	Low
operon_142	+	Spy49_0230, Spy49_0231	238829	High

operon_143	+	Spy49_0233	240042	Low
operon_144	+	Spy49_0234	242541	High
operon_145	-	Spy49_0235c	243840	High
operon_146	-	Spy49_0236c, Spy49_0237c	244142	High
operon_147	+	Spy49_0238	246636	High
operon_148	+	Spy49_0239	248455	High
operon_149	+	Spy49_0240, Spy49_0241	249601	High
operon_150	+	Spy49_0242, Spy49_0243, Spy49_0244, Spy49_0245, Spy49_0246	251605	High
operon_151	-	Spy49_0247	257083	High
operon_152	-	Spy49_0248c	258595	High
operon_153	+	Spy49_0249, Spy49_0250, Spy49_0251, Spy49_0252, Spy49_0253	259952	High
operon_154	-	Spy49_0254c	266666	High
operon_155	+	Spy49_rRNA16s4	267459	High
operon_156	+	Spy49_tRNAala2, Spy49_tRNAala6	269048	High
operon_157	+	Spy49_rRNA23s4	269556	High
operon_158	+	Spy49_tRNAasn1, Spy49_tRNAasn2, Spy49_tRNAarg2	272517	High
operon_159	+	Spy49_0256	272782	High
operon_160	+	Spy49_0257, Spy49_0258, Spy49_0259, Spy49_0260, Spy49_0261, Spy49_0262, Spy49_0263	273880	High
operon_161	+	Spy49_0264	278447	Low
operon_162	+	Spy49_0265	280082	High
operon_163	+	Spy49_0266	281049	High
operon_164	+	Spy49_0268	281916	Low
operon_165	+	Spy49_0269, Spy49_0270	283204	Low
operon_166	-	Spy49_0271c	285113	Low
operon_167	+	Spy49_0272	286647	Low
operon_168	-	Spy49_0273c, Spy49_0274c	288009	High
operon_169	-	Spy49_0275	290144	High
operon_170	+	Spy49_0276, Spy49_0277	290994	High
operon_171	+	Spy49_0278	292519	Low
operon_172	+	Spy49_0279	293366	High
operon_173	+	Spy49_0280	294422	Low
operon_174	+	Spy49_0281, Spy49_0282, Spy49_0283, Spy49_0284	295857	Low
operon_175	+	Spy49_0285	299936	Low
operon_176	+	Spy49_0286, Spy49_0287, Spy49_0288	303417	High
operon_177	+	Spy49_0289	305999	Low
operon_178	+	Spy49_0290	307639	Low

operon_179	-	Spy49_0291, Spy49_0292c	308750	High
operon_180	+	Spy49_0293	310346	High
operon_181	+	Spy49_0294, Spy49_0295	311101	Low
operon_182	+	Spy49_0296	312483	High
operon_183	+	Spy49_0297, Spy49_0298, Spy49_0299, Spy49_0300, Spy49_0301, Spy49_0302, Spy49_0303	312833	Low
operon_184	+	Spy49_0304, Spy49_0305	317713	Low
operon_185	+	Spy49_0306	318833	High
operon_186	+	Spy49_0307	319594	High
operon_187	+	Spy49_0308	320371	High
operon_188	+	Spy49_0309, Spy49_0310	321157	Low
operon_189	+	Spy49_0311	322883	Low
operon_190	+	Spy49_0312	324331	High
operon_191	+	Spy49_0313	325219	Low
operon_192	+	Spy49_0314	326304	High
operon_193	-	Spy49_0315, Spy49_0316, Spy49_0317c, Spy49_0318	326985	High
operon_194	-	Spy49_0319c	330920	High
operon_195	+	Spy49_0320	332526	High
operon_196	+	Spy49_0322	334331	High
operon_197	+	Spy49_0323	335094	Low
operon_198	+	Spy49_0325	336056	High
operon_199	+	Spy49_0326, Spy49_0327	336688	Low
operon_200	+	Spy49_0328	338514	Low
operon_201	+	Spy49_0329, Spy49_0330, Spy49_0331	338927	High
operon_202	-	Spy49_0332c	340644	High
operon_203	+	Spy49_0333	341514	High
operon_204	-	Spy49_0334c	341999	High
operon_205	+	Spy49_0335	343044	High
operon_206	+	Spy49_0336	344290	Low
operon_207	+	Spy49_0337	349721	High
operon_208	+	Spy49_0338	351062	High
operon_209	+	Spy49_0339, Spy49_0340, Spy49_0341	353439	High
operon_210	-	Spy49_0342c	357478	High
operon_211	+	Spy49_0343, Spy49_0344	358497	High
operon_212	-	Spy49_0345c	359497	Low
operon_213	-	Spy49_0346c	359896	Low
operon_214	+	Spy49_0347	360825	High
operon_215	+	Spy49_0348	361520	High
operon_216	-	Spy49_0349c	362815	High
operon_217	-	Spy49_0350c	364170	High

operon_218	-	Spy49_0351c	365111	Low
operon_219	-	Spy49_0352c, Spy49_0353c, Spy49_0354c	365504	High
operon_220	-	Spy49_0355c	366567	Low
operon_221	-	Spy49_0356c, Spy49_0357c, Spy49_0358c	367138	High
operon_222	-	Spy49_0359c	368661	High
operon_223	-	Spy49_0360c	369225	Low
operon_224	-	Spy49_0361c, Spy49_0362c	369632	Low
operon_225	-	Spy49_0363c, Spy49_0364c	372362	Low
operon_226	-	Spy49_0365c, Spy49_0366c	372942	Low
operon_227	-	Spy49_0367c, Spy49_0368c	373864	High
operon_228	+	Spy49_0369, Spy49_0370	374588	High
operon_229	+	Spy49_0371, Spy49_0372, Spy49_0373	376907	High
operon_230	+	Spy49_0374	379624	High
operon_231	+	Spy49_0375, Spy49_0376, Spy49_0377, Spy49_0378	381081	Low
operon_232	+	Spy49_0379	384176	Low
operon_233	-	Spy49_0380c	384646	High
operon_234	+	Spy49_0381	385442	High
operon_235	+	Spy49_0382, Spy49_0383	386589	High
operon_236	-	Spy49_0384c	388187	High
operon_237	+	Spy49_0385	389233	High
operon_238	-	Spy49_0386c	391667	High
operon_239	+	Spy49_0387	392224	High
operon_240	+	Spy49_0388	392779	Low
operon_241	+	Spy49_0390, Spy49_0391	393828	High
operon_242	+	Spy49_0392	395212	Low
operon_243	+	Spy49_0394, Spy49_0395	396175	High
operon_244	+	Spy49_0396	397056	High
operon_245	+	Spy49_0398	398438	High
operon_246	+	Spy49_0399, Spy49_0400	400465	High
operon_247	+	Spy49_0401, Spy49_0402	402240	Low
operon_248	+	Spy49_0403, Spy49_0404	403264	Low
operon_249	-	Spy49_0405c	404797	Low
operon_250	+	Spy49_0406	405518	High
operon_251	-	Spy49_0407c, Spy49_0408c	406412	High
operon_252	+	Spy49_0409	407157	High
operon_253	+	Spy49_0410	407919	Low
operon_254	+	Spy49_0411	408663	Low
operon_255	+	Spy49_0412, Spy49_0413	409108	High
operon_256	-	Spy49_0414c	411330	High
operon_257	+	Spy49_0415	412646	High
operon_258	+	Spy49_0416, Spy49_0417	413625	Low

operon_259	+	Spy49_0418	415246	High
operon_260	+	Spy49_0419, Spy49_0420	416881	Low
operon_261	+	Spy49_0421	418315	High
operon_262	+	Spy49_0422, Spy49_0423, Spy49_0424	418607	Low
operon_263	-	Spy49_0425c, Spy49_0426c, Spy49_0427c	422274	High
operon_264	-	Spy49_0428c	424713	Low
operon_265	-	Spy49_0429c, Spy49_0430c, Spy49_0431c	425877	High
operon_266	+	Spy49_0432	428212	High
operon_267	+	Spy49_0433, Spy49_0434	429387	High
operon_268	+	Spy49_0435	431934	High
operon_269	+	Spy49_0436, Spy49_0437, Spy49_0438	434187	High
operon_270	+	Spy49_0439	436922	Low
operon_271	+	Spy49_0440, Spy49_0441	437536	High
operon_272	+	Spy49_0442	439999	High
operon_273	+	Spy49_0444, Spy49_0445, Spy49_0446	441089	Low
operon_274	+	Spy49_0447, Spy49_0448	444459	High
operon_275	-	Spy49_0449c	448862	High
operon_276	+	Spy49_0450, Spy49_0451, Spy49_0453, Spy49_0454, Spy49_0455, Spy49_0456	449874	High
operon_277	+	Spy49_0457	455664	Low
operon_278	+	Spy49_0458, Spy49_0459, Spy49_0460	456915	Low
operon_279	-	Spy49_0461c, Spy49_0462c, Spy49_0463c, Spy49_0464c	459944	High
operon_280	+	Spy49_0465	462941	Low
operon_281	+	Spy49_0466	463161	High
operon_282	+	Spy49_0467	463538	Low
operon_283	+	Spy49_0468, Spy49_0469	464207	High
operon_284	-	Spy49_0470c	465077	High
operon_285	+	Spy49_0471, Spy49_0473	465422	Low
operon_286	+	Spy49_0474	466537	Low
operon_287	+	Spy49_0475	467978	Low
operon_288	+	Spy49_0476	468896	Low
operon_289	-	Spy49_0477c, Spy49_0478c	470066	Low
operon_290	+	Spy49_0479	471556	Low
operon_291	+	Spy49_0480, Spy49_0481, Spy49_0482	471646	High
operon_292	-	Spy49_0483c	474951	High
operon_293	+	Spy49_0484, Spy49_0485, Spy49_0487	476512	High
operon_294	-	Spy49_0488c, Spy49_0489c	480912	High
operon_295	+	Spy49_0490, Spy49_0491	483169	High
operon_296	+	Spy49_0493	485770	High
operon_297	+	Spy49_0494, Spy49_0495, Spy49_0496, Spy49_0497	486410	High

operon_298	+	Spy49_0498	489015	High
operon_299	+	Spy49_0499	489297	High
operon_300	+	Spy49_0500	490608	Low
operon_301	+	Spy49_0501	492081	High
operon_302	-	Spy49_0502c	492448	High
operon_303	-	Spy49_0503c	492691	High
operon_304	+	Spy49_0504	494253	Low
operon_305	-	Spy49_0505c	495345	Low
operon_306	-	Spy49_0506c	496217	Low
operon_307	-	Spy49_0507c	496963	High
operon_308	-	Spy49_0508c	497786	High
operon_309	+	Spy49_0509, Spy49_0510	498946	High
operon_310	-	Spy49_0511c, Spy49_0512c	499867	High
operon_311	+	Spy49_0513	502319	High
operon_312	+	Spy49_0514	505257	High
operon_313	+	Spy49_0515	506846	High
operon_314	+	Spy49_0516	508327	High
operon_315	-	Spy49_0517c, Spy49_0518c, Spy49_0519c	509144	High
operon_316	-	Spy49_0520c	512653	High
operon_317	+	Spy49_0521	514281	High
operon_318	+	Spy49_0522	514805	High
operon_319	-	Spy49_0523c	517658	High
operon_320	-	Spy49_0524c	518714	Low
operon_321	-	Spy49_0525c, Spy49_0526c, Spy49_0527c, Spy49_0528c, Spy49_0529c	520697	Low
operon_322	+	Spy49_0530, Spy49_0531, Spy49_0532, Spy49_0533	524813	High
operon_323	+	Spy49_0534	528259	High
operon_324	+	Spy49_0535	529270	Low
operon_325	+	Spy49_0536, Spy49_0537, Spy49_0538	530835	Low
operon_326	-	Spy49_0539c	533766	High
operon_327	+	Spy49_0540	534629	High
operon_328	+	Spy49_0541	535536	Low
operon_329	+	Spy49_0543, Spy49_0544	538259	Low
operon_330	+	Spy49_0545, Spy49_0546, Spy49_0547	541377	High
operon_331	+	Spy49_0548	544286	High
operon_332	+	Spy49_0549	545779	Low
operon_333	+	Spy49_0550, Spy49_0551	547528	High
operon_334	-	Spy49_0552c	549567	High
operon_335	-	Spy49_0554c	549908	Low
operon_336	+	Spy49_0555	551283	High
operon_337	+	Spy49_0556, Spy49_0557	551747	Low

operon_338	+	Spy49_0558	553502	High
operon_339	+	Spy49_tRNAarg4	554162	High
operon_340	+	Spy49_0559	554960	High
operon_341	+	Spy49_0560	555517	Low
operon_342	+	Spy49_0561	557653	Low
operon_343	-	Spy49_0562c	559627	Low
operon_344	+	Spy49_0563	560358	High
operon_345	-	Spy49_0564c, Spy49_0565c	562294	High
operon_346	-	Spy49_0566c	563283	Low
operon_347	+	Spy49_0567	565244	High
operon_348	+	Spy49_0568	569653	High
operon_349	+	Spy49_0569, Spy49_0570, Spy49_0572, Spy49_0573, Spy49_0574, Spy49_0575, Spy49_0576, Spy49_0577	570093	Low
operon_350	+	Spy49_0578	578384	Low
operon_351	+	Spy49_0579	581587	High
operon_352	+	Spy49_0580, Spy49_0581	582384	High
operon_353	+	Spy49_0582, Spy49_0583, Spy49_0584, Spy49_0585, Spy49_0586, Spy49_0587	585413	High
operon_354	+	Spy49_0588, Spy49_0589	590254	Low
operon_355	+	Spy49_0590, Spy49_0591, Spy49_0592	592303	Low
operon_356	+	Spy49_0596	594649	Low
operon_357	+	Spy49_0597	596225	Low
operon_358	+	Spy49_0598, Spy49_0599, Spy49_0600, Spy49_0601	598783	High
operon_359	-	Spy49_0602c	601517	High
operon_360	+	Spy49_0603, Spy49_0604	602574	High
operon_361	+	Spy49_0605	609514	Low
operon_362	+	Spy49_0606	610160	High
operon_363	-	Spy49_0607c	610762	High
operon_364	+	Spy49_0608, Spy49_0609	611338	High
operon_365	+	Spy49_0610	614389	Low
operon_366	+	Spy49_0611	614914	Low
operon_367	+	Spy49_0612, Spy49_0613, Spy49_0614, Spy49_0615, Spy49_0617, Spy49_0618, Spy49_0619	615940	High
operon_368	+	Spy49_0620, Spy49_0621, Spy49_0622, Spy49_0623	625379	Low
operon_369	+	Spy49_0624, Spy49_0625	629341	High
operon_370	-	Spy49_0626c	631102	Low
operon_371	+	Spy49_0627, Spy49_0628	631317	Low
operon_372	+	Spy49_0629, Spy49_0630, Spy49_0631	632593	High

operon_373	-	Spy49_0632c	634124	High
operon_374	+	Spy49_0633, Spy49_0634, Spy49_0635, Spy49_0636	636474	High
operon_375	+	Spy49_0637	640008	Low
operon_376	-	Spy49_0638c, Spy49_0639c	641624	High
operon_377	+	Spy49_0640, Spy49_0641	643592	High
operon_378	+	Spy49_0642	646097	High
operon_379	+	Spy49_0643, Spy49_0644, Spy49_0645	647259	High
operon_380	+	Spy49_0646, Spy49_0647, Spy49_0648	648827	High
operon_381	+	Spy49_0650, Spy49_0651, Spy49_0652, Spy49_0653	651349	High
operon_382	+	Spy49_0654	655533	Low
operon_383	+	Spy49_0655, Spy49_0656, Spy49_0657	659019	High
operon_384	+	Spy49_0658	662381	Low
operon_385	+	Spy49_0659, Spy49_0660	664308	High
operon_386	+	Spy49_0661, Spy49_0662	665338	High
operon_387	-	Spy49_0663c	669334	Low
operon_388	+	Spy49_0664	670391	High
operon_389	+	Spy49_0665, Spy49_0667, Spy49_0668	671105	High
operon_390	+	Spy49_0669, Spy49_0671	673556	High
operon_391	+	Spy49_0672, Spy49_0673, Spy49_0674	675798	High
operon_392	+	Spy49_0675	679492	High
operon_393	+	Spy49_0676	680230	High
operon_394	-	Spy49_0677c	681030	Low
operon_395	-	Spy49_0679	681584	Low
operon_396	+	Spy49_0680, Spy49_0681	683698	High
operon_397	+	Spy49_0682, Spy49_0683	685237	High
operon_398	+	Spy49_0684	688727	High
operon_399	-	Spy49_0686c	689631	Low
operon_400	+	Spy49_0687, Spy49_0688, Spy49_0689	691904	High
operon_401	+	Spy49_0690, Spy49_0691, Spy49_0692, Spy49_0693	694731	High
operon_402	-	Spy49_0694c, Spy49_0695c	698743	High
operon_403	+	Spy49_0696, Spy49_0697, Spy49_0698, Spy49_0700, Spy49_0701	701396	High
operon_404	+	Spy49_0702	705012	Low
operon_405	-	Spy49_0703c	705948	High
operon_406	+	Spy49_0704, Spy49_0705, Spy49_0706, Spy49_0707	708396	High
operon_407	+	Spy49_0708	711912	Low
operon_408	+	Spy49_0709	712943	Low
operon_409	-	Spy49_0710c	713767	Low

operon_410	+	Spy49_0711	714895	High
operon_411	+	Spy49_0712, Spy49_0713	715637	Low
operon_412	+	Spy49_0714	717225	Low
operon_413	+	Spy49_0715, Spy49_0716	718836	Low
operon_414	+	Spy49_0717	720525	High
operon_415	+	Spy49_0718	721289	Low
operon_416	-	Spy49_0719c	722677	High
operon_417	+	Spy49_0720, Spy49_0721	723438	High
operon_418	+	Spy49_0722	727961	Low
operon_419	+	Spy49_0723	729160	High
operon_420	+	Spy49_tRNAgln1	729430	Low
operon_421	+	Spy49_0724	729666	High
operon_422	+	Spy49_tRNAarg5	731036	High
operon_423	-	Spy49_0725c	731205	High
operon_424	+	Spy49_0726	731978	High
operon_425	+	Spy49_0727	732991	Low
operon_426	+	Spy49_0728	733590	High
operon_427	-	Spy49_0729c	734955	High
operon_428	+	Spy49_0730, Spy49_0731, Spy49_0732, Spy49_0733	735215	High
operon_429	+	Spy49_0734, Spy49_0735	739180	High
operon_430	+	Spy49_0736, Spy49_0737, Spy49_0738	742115	Low
operon_431	+	Spy49_0739, Spy49_0740, Spy49_0741	744176	High
operon_432	+	Spy49_0742, Spy49_0744	746848	High
operon_433	+	Spy49_0745	748448	Low
operon_434	-	Spy49_0746c	749661	High
operon_435	-	Spy49_0747c, Spy49_0748c, Spy49_0749c	750936	High
operon_436	+	Spy49_0750	752165	Low
operon_437	-	Spy49_0751c	752456	Low
operon_438	+	Spy49_0752, Spy49_0753, Spy49_0754, Spy49_0755, Spy49_0756	753283	Low
operon_439	+	Spy49_0757	754713	Low
operon_440	+	Spy49_0759, Spy49_0760, Spy49_0761, Spy49_0762, Spy49_0763	755031	High
operon_441	+	Spy49_0764, Spy49_0765	761705	High
operon_442	+	Spy49_0766	763521	Low
operon_443	+	Spy49_0767	763942	Low
operon_444	+	Spy49_0768, Spy49_0769, Spy49_0770, Spy49_0771	764484	High
operon_445	+	Spy49_0772, Spy49_0773, Spy49_0774, Spy49_0775, Spy49_0776, Spy49_0777, Spy49_0778, Spy49_0779	769300	Low

operon_446	+	Spy49_0780, Spy49_0781, Spy49_0782, Spy49_0783, Spy49_0784, Spy49_0785, Spy49_0786, Spy49_0788, Spy49_0789	773340	Low
operon_447	+	Spy49_0790	784893	Low
operon_448	+	Spy49_0791	785820	High
operon_449	+	Spy49_0792	786260	Low
operon_450	+	Spy49_0793, Spy49_0794, Spy49_0795	787527	High
operon_451	-	Spy49_0796c	790666	High
operon_452	+	Spy49_0797, Spy49_0798	792442	High
operon_453	+	Spy49_0799, Spy49_0800	793949	Low
operon_454	+	Spy49_0801	795789	High
operon_455	+	Spy49_0803, Spy49_0804	797593	Low
operon_456	+	Spy49_0805	798815	Low
operon_457	+	Spy49_0807, Spy49_0808	801023	High
operon_458	+	Spy49_0809	803156	Low
operon_459	+	Spy49_0810	804821	Low
operon_460	-	Spy49_0811c	807144	High
operon_461	+	Spy49_0812	810046	High
operon_462	-	Spy49_0813c, Spy49_0814c	811189	High
operon_463	+	Spy49_0815, Spy49_0816, Spy49_0817	813387	High
operon_464	+	Spy49_0818, Spy49_0819, Spy49_0820, Spy49_0821, Spy49_0822	816640	Low
operon_465	+	Spy49_0823, Spy49_0825, Spy49_0826, Spy49_0827	821068	High
operon_466	+	Spy49_0828	827564	High
operon_467	+	Spy49_0829	828208	High
operon_468	+	Spy49_0830	830197	Low
operon_469	+	Spy49_0831	831598	High
operon_470	+	Spy49_0832	832178	High
operon_471	+	Spy49_0833, Spy49_0834	833334	High
operon_472	+	Spy49_0835, Spy49_0836	834410	High
operon_473	+	Spy49_0837, Spy49_0839, Spy49_0840	836143	High
operon_474	+	Spy49_0841, Spy49_0842	839725	High
operon_475	+	Spy49_0843	843128	Low
operon_476	+	Spy49_0844	843886	Low
operon_477	-	Spy49_0845c	845398	High
operon_478	+	Spy49_0846, Spy49_0847, Spy49_0848	846969	High
operon_479	+	Spy49_0849	848301	High
operon_480	-	Spy49_0850c	849608	High
operon_481	+	Spy49_0851, Spy49_0852, Spy49_0853, Spy49_0854, Spy49_0855	850934	High

operon_482	+	Spy49_0856, Spy49_0857, Spy49_0858, Spy49_0859, Spy49_0860	854618	High
operon_483	-	Spy49_0861c, Spy49_0862c	859407	High
operon_484	+	Spy49_0863, Spy49_0864	861630	Low
operon_485	-	Spy49_0865c	864373	High
operon_486	+	Spy49_0866	865656	High
operon_487	+	Spy49_0867	866563	High
operon_488	+	Spy49_0868	868196	Low
operon_489	+	Spy49_0869	869065	High
operon_490	+	Spy49_0870	870212	High
operon_491	-	Spy49_0871c	870928	Low
operon_492	-	Spy49_0872c	871529	High
operon_493	-	Spy49_0873c, Spy49_0874c, Spy49_0875c	872404	Low
operon_494	-	Spy49_0876c	875049	Low
operon_495	+	Spy49_0877, Spy49_0878, Spy49_0879	875731	High
operon_496	+	Spy49_0880	878163	High
operon_497	+	Spy49_0881	879234	Low
operon_498	-	Spy49_0882c	880138	Low
operon_499	+	Spy49_0883, Spy49_0884	881137	Low
operon_500	+	Spy49_0885	883737	High
operon_501	-	Spy49_0886c	884877	Low
operon_502	+	Spy49_0887, Spy49_0888	885158	Low
operon_503	-	Spy49_0891c, Spy49_0892c	887052	High
operon_504	+	Spy49_0893, Spy49_0894, Spy49_0895, Spy49_0896, Spy49_0897, Spy49_0898, Spy49_0899, Spy49_0900, Spy49_0901, Spy49_0902	888381	High
operon_505	+	Spy49_0903	898472	High
operon_506	-	Spy49_0904c	900023	High
operon_507	+	Spy49_0905, Spy49_0906	901180	High
operon_508	+	Spy49_0907	904461	Low
operon_509	+	Spy49_0908	905182	High
operon_510	-	Spy49_0909c	906058	High
operon_511	-	Spy49_0910c, Spy49_0911c	907094	Low
operon_512	+	Spy49_0912, Spy49_0913	908312	High
operon_513	+	Spy49_0914	910022	Low
operon_514	+	Spy49_0915	911008	High
operon_515	-	Spy49_0916c, Spy49_0917c	913213	High
operon_516	+	Spy49_0918, Spy49_0919	913987	Low
operon_517	+	Spy49_0920, Spy49_0921	915796	High
operon_518	+	Spy49_0922	917274	High

operon_519	+	Spy49_0923, Spy49_0924, Spy49_0925, Spy49_0926	918827	High
operon_520	-	Spy49_0927c, Spy49_0928c	922186	Low
operon_521	-	Spy49_0929c	923990	High
operon_522	+	Spy49_0930	925766	High
operon_523	+	Spy49_0933	926367	Low
operon_524	+	Spy49_0934, Spy49_0936, Spy49_0937, Spy49_0938, Spy49_0939	927826	Low
operon_525	-	Spy49_0940c	932716	High
operon_526	-	Spy49_0942c	933875	Low
operon_527	-	Spy49_0943c	934698	Low
operon_528	+	Spy49_0944	936569	High
operon_529	-	Spy49_0945c	937395	High
operon_530	-	Spy49_0946c	938970	Low
operon_531	-	Spy49_0947c	939433	High
operon_532	-	Spy49_0948c	940147	Low
operon_533	+	Spy49_0949	941752	High
operon_534	-	Spy49_0951c	943409	High
operon_535	-	Spy49_0952c	944861	High
operon_536	-	Spy49_0953c, Spy49_0954c	946552	High
operon_537	+	Spy49_0955	948115	High
operon_538	-	Spy49_0957c	949822	High
operon_539	-	Spy49_0958c, Spy49_0959c	950391	High
operon_540	-	Spy49_0960c, Spy49_0961c, Spy49_0962c, Spy49_0963c, Spy49_0964c, Spy49_0965c, Spy49_0966c	953941	Low
operon_541	+	Spy49_0967, Spy49_0968	960356	High
operon_542	+	Spy49_0969	961621	High
operon_543	+	Spy49_0970	962293	Low
operon_544	-	Spy49_0971c, Spy49_0972c, Spy49_0973c	964170	High
operon_545	-	Spy49_0974c, Spy49_0975c	967972	High
operon_546	-	Spy49_0976c	970002	Low
operon_547	+	Spy49_0977	971027	High
operon_548	+	Spy49_0978	972013	High
operon_549	-	Spy49_0979c, Spy49_0980c	972380	High
operon_550	-	Spy49_0981c	974709	High
operon_551	-	Spy49_0982c, Spy49_0983c, Spy49_0984c, Spy49_0985c, Spy49_0986, Spy49_0987c	977358	Low
operon_552	-	Spy49_0988c, Spy49_0989c, Spy49_0990c	982531	Low
operon_553	-	Spy49_0991c	984960	High
operon_554	-	Spy49_0992c, Spy49_0993c	985394	Low
operon_555	-	Spy49_0994c	987321	Low

operon_556	-	Spy49_0995c, Spy49_0996c, Spy49_0997c, Spy49_0998c	988785	High
operon_557	+	Spy49_0999	993855	Low
operon_558	-	Spy49_1000c	994721	High
operon_559	-	Spy49_1001c, Spy49_1002c, Spy49_1003c, Spy49_1004c, Spy49_1005c, Spy49_1006c	995384	High
operon_560	-	Spy49_1007c	998161	High
operon_561	+	Spy49_1008	1000763	High
operon_562	+	Spy49_1009	1002461	High
operon_563	-	Spy49_1010c	1003826	High
operon_564	-	Spy49_1011c, Spy49_1012c, Spy49_1013c	1005216	High
operon_565	-	Spy49_1014c	1007403	Low
operon_566	-	Spy49_1015c	1007961	High
operon_567	-	Spy49_1016c	1009988	High
operon_568	-	Spy49_1017c, Spy49_1018c	1010730	High
operon_569	-	Spy49_1019c	1013350	Low
operon_570	+	Spy49_1020, Spy49_1021, Spy49_1022	1016677	High
operon_571	-	Spy49_1023c	1018678	High
operon_572	-	Spy49_tmRNA	1019392	High
operon_573	+	Spy49_1024	1020025	High
operon_574	-	Spy49_1025c, Spy49_1026c	1020769	High
operon_575	-	Spy49_1027c	1024834	High
operon_576	+	Spy49_1028	1026066	High
operon_577	+	Spy49_1029, Spy49_1030	1027540	High
operon_578	+	Spy49_1819c	1029995	High
operon_579	+	Spy49_1032	1031212	High
operon_580	-	Spy49_1033c, Spy49_1034c, Spy49_1035c, Spy49_1036c, Spy49_1037c, Spy49_1038c	1031324	High
operon_581	-	Spy49_1039c	1037216	High
operon_582	+	Spy49_1040, Spy49_1041	1039382	High
operon_583	-	Spy49_1042c, Spy49_1043c	1042457	High
operon_584	-	Spy49_1044c, Spy49_1045c, Spy49_1046c, Spy49_1047c	1044082	Low
operon_585	-	Spy49_1048c, Spy49_1049c	1047336	Low
operon_586	+	Spy49_1050	1050117	High
operon_587	-	Spy49_1051c, Spy49_1053c	1050815	Low
operon_588	+	Spy49_1054	1052822	High
operon_589	+	Spy49_1055	1053308	High
operon_590	+	Spy49_1056	1054134	High
operon_591	+	Spy49_1057	1055088	High
operon_592	+	Spy49_1059	1057164	High
operon_593	+	Spy49_1060, Spy49_1061c	1058692	Low

operon_594	+	Spy49_1062	1058918	High
operon_595	-	Spy49_1064c	1059832	Low
operon_596	+	Spy49_1065	1060583	High
operon_597	-	Spy49_1066c	1061023	High
operon_598	+	Spy49_1067	1062674	High
operon_599	-	Spy49_1070c	1064238	High
operon_600	-	Spy49_1071c	1064544	High
operon_601	-	Spy49_1072c	1065479	Low
operon_602	-	Spy49_1073c	1067108	High
operon_603	-	Spy49_1074c, Spy49_1075c	1068683	Low
operon_604	-	Spy49_1076c, Spy49_1077c, Spy49_1078c, Spy49_1079c	1070610	Low
operon_605	-	Spy49_1080c	1074476	Low
operon_606	-	Spy49_1082c	1075497	High
operon_607	-	Spy49_1083c	1076927	High
operon_608	-	Spy49_1084c	1078613	High
operon_609	+	Spy49_1085	1081295	High
operon_610	-	Spy49_1086c, Spy49_1087c, Spy49_1088c	1082183	Low
operon_611	+	Spy49_1089	1084854	High
operon_612	-	Spy49_1090c	1085972	High
operon_613	+	Spy49_1091	1086687	High
operon_614	-	Spy49_1092c	1087895	High
operon_615	-	Spy49_1093c	1089263	High
operon_616	-	Spy49_1094c, Spy49_1095c	1090880	High
operon_617	+	Spy49_1096	1093250	High
operon_618	+	Spy49_1097, Spy49_1098, Spy49_1099	1093514	Low
operon_619	+	Spy49_1100	1096984	Low
operon_620	+	Spy49_1101	1098332	Low
operon_621	+	Spy49_1103, Spy49_1104	1098845	High
operon_622	-	Spy49_1105c, Spy49_1106c, Spy49_1107c	1099605	Low
operon_623	-	Spy49_1108c	1101678	High
operon_624	-	Spy49_1109c	1104511	Low
operon_625	-	Spy49_1110c	1105791	Low
operon_626	-	Spy49_1111c	1106549	High
operon_627	-	Spy49_1112c	1108117	High
operon_628	-	Spy49_1113c	1109988	Low
operon_629	-	Spy49_1114c	1111097	Low
operon_630	-	Spy49_1115c	1112021	Low
operon_631	+	Spy49_1116, Spy49_1117	1112936	High
operon_632	-	Spy49_1119c, Spy49_1120c, Spy49_1121c	1115287	High
operon_633	-	Spy49_1122c	1116543	High
operon_634	-	Spy49_1123c	1117217	Low

operon_635	-	Spy49_1124c, Spy49_1126c	1118341	Low
operon_636	-	Spy49_1127c	1121421	High
operon_637	+	Spy49_1128, Spy49_1129	1122321	High
operon_638	-	Spy49_1130c	1123386	High
operon_639	-	Spy49_1132c	1125493	High
operon_640	-	Spy49_1133c	1127289	Low
operon_641	-	Spy49_1135c, Spy49_1136c	1129193	High
operon_642	-	Spy49_1137c, Spy49_1138c, Spy49_1139c	1131434	Low
operon_643	-	Spy49_1140c	1134121	High
operon_644	-	Spy49_1141c	1134986	High
operon_645	-	Spy49_1142c	1135589	Low
operon_646	-	Spy49_1143c	1136360	High
operon_647	+	Spy49_1144	1137360	High
operon_648	-	Spy49_1145c, Spy49_1146c	1138286	High
operon_649	-	Spy49_1147c	1140699	High
operon_650	-	Spy49_1148c, Spy49_1149c, Spy49_1150c	1141043	Low
operon_651	-	Spy49_1151c	1143465	Low
operon_652	-	Spy49_1152c, Spy49_1153c, Spy49_1154c	1144615	Low
operon_653	-	Spy49_1155c, Spy49_1156c, Spy49_1157c	1147869	High
operon_654	-	Spy49_1158c	1150182	High
operon_655	-	Spy49_1159c	1151218	High
operon_656	-	Spy49_1160c	1153047	Low
operon_657	-	Spy49_1161c, Spy49_1162c	1154692	High
operon_658	-	Spy49_1163c	1156235	High
operon_659	+	Spy49_1164	1156719	High
operon_660	+	Spy49_1165, Spy49_1166	1159172	High
operon_661	-	Spy49_1167c	1160219	High
operon_662	-	Spy49_1168c, Spy49_1169c, Spy49_1170c, Spy49_1171c, Spy49_1172c, Spy49_1173c, Spy49_1174c	1163136	High
operon_663	-	Spy49_1175c, Spy49_1176c, Spy49_1177c	1169318	High
operon_664	-	Spy49_1178c	1173314	High
operon_665	-	Spy49_1179c	1173677	Low
operon_666	-	Spy49_1180c, Spy49_1181c	1175671	Low
operon_667	-	Spy49_1182c	1177135	Low
operon_668	-	Spy49_1183c	1177452	High
operon_669	+	Spy49_1184	1178173	Low
operon_670	-	Spy49_1185c, Spy49_1186c	1178901	High
operon_671	-	Spy49_1187c	1180872	Low
operon_672	-	Spy49_1188c, Spy49_1189c, Spy49_1190c	1182436	High
operon_673	-	Spy49_1191c	1184593	Low
operon_674	-	Spy49_1192c, Spy49_1193c, Spy49_1194c	1185810	High

operon_675	-	Spy49_1195c, Spy49_1196c, Spy49_1197c	1189653	High
operon_676	-	Spy49_1198c	1193025	High
operon_677	+	Spy49_1199	1193828	High
operon_678	-	Spy49_1200c, Spy49_1201c, Spy49_1202c, Spy49_1203c	1194453	High
operon_679	-	Spy49_1204c, Spy49_1205c, Spy49_1206c	1198887	Low
operon_680	-	Spy49_1207c, Spy49_1208c, Spy49_1209c, Spy49_1210c, Spy49_1211c, Spy49_1212c	1201583	High
operon_681	-	Spy49_1213c	1207642	Low
operon_682	-	Spy49_1215c	1210266	High
operon_683	-	Spy49_1216c, Spy49_1217c	1212919	Low
operon_684	-	Spy49_1218c, Spy49_1219c	1213977	High
operon_685	+	Spy49_tRNAthr3	1214924	High
operon_686	+	Spy49_1220, Spy49_1221	1215135	High
operon_687	-	Spy49_1222c, Spy49_1223c, Spy49_1224c	1217340	Low
operon_688	-	Spy49_1225c	1219195	Low
operon_689	-	Spy49_1226c, Spy49_1227c, Spy49_1228c, Spy49_1229c	1220183	Low
operon_690	-	Spy49_1230c, Spy49_1231c, Spy49_1233c	1227791	High
operon_691	+	Spy49_1234	1231307	Low
operon_692	-	Spy49_1235c, Spy49_1236c, Spy49_1237c	1232843	High
operon_693	+	Spy49_1238, Spy49_1239	1237255	High
operon_694	-	Spy49_1240c	1241346	High
operon_695	-	Spy49_1241c	1243212	High
operon_696	+	Spy49_1242	1244876	High
operon_697	+	Spy49_1243	1245691	Low
operon_698	+	Spy49_1244, Spy49_1245	1246530	Low
operon_699	-	Spy49_tRNAile1, Spy49_tRNAile3	1247348	High
operon_700	-	Spy49_tRNAthr4, Spy49_tRNAleu3	1247596	High
operon_701	-	Spy49_tRNAval1, Spy49_tRNAval2, Spy49_tRNAval3	1247891	High
operon_702	-	Spy49_rRNA5s4	1248021	High
operon_703	-	Spy49_rRNA23s5	1248223	High
operon_704	-	Spy49_tRNAala5	1250995	High
operon_705	-	Spy49_rRNA16s2	1251542	High
operon_706	-	Spy49_1247c	1253757	High
operon_707	-	Spy49_1248c, Spy49_1249c	1254594	Low
operon_708	+	Spy49_1250	1256646	High
operon_709	+	Spy49_1251	1257372	High
operon_710	-	Spy49_1252c, Spy49_1253c, Spy49_1254c, Spy49_1255c, Spy49_1256c	1258369	High

operon_711	-	Spy49_1257c, Spy49_1259c, Spy49_1260c, Spy49_1261c	1262620	High
operon_712	-	Spy49_1262c	1267580	Low
operon_713	-	Spy49_1263c, Spy49_1265c	1270056	High
operon_714	-	Spy49_1266c	1271125	High
operon_715	-	Spy49_1267c	1272784	High
operon_716	+	Spy49_1268, Spy49_1269, Spy49_1270, Spy49_1271, Spy49_1272	1273872	Low
operon_717	+	Spy49_1273	1278768	High
operon_718	-	Spy49_1274c, Spy49_1275c	1279394	High
operon_719	-	Spy49_RNPA	1282084	High
operon_720	-	Spy49_1276c	1282490	Low
operon_721	-	Spy49_1277c	1282870	Low
operon_722	+	Spy49_1278	1283507	High
operon_723	+	Spy49_1279	1284139	Low
operon_724	-	Spy49_1280c	1286632	High
operon_725	-	Spy49_1281c, Spy49_1282c	1288257	High
operon_726	-	Spy49_1283c	1290712	High
operon_727	-	Spy49_1284c	1292255	High
operon_728	-	Spy49_1285c	1293234	Low
operon_729	-	Spy49_1286c, Spy49_1287c	1293600	High
operon_730	-	Spy49_1289c	1295238	Low
operon_731	-	Spy49_1290c, Spy49_1291c, Spy49_1292c, Spy49_1293c	1296788	High
operon_732	-	Spy49_1294c, Spy49_1295c, Spy49_1296c	1301475	High
operon_733	-	Spy49_1297c, Spy49_1298c, Spy49_1299c	1304019	High
operon_734	-	Spy49_1300c	1306882	High
operon_735	-	Spy49_1301c	1309098	High
operon_736	-	Spy49_1303c, Spy49_1304c	1309830	Low
operon_737	-	Spy49_1305c, Spy49_1306c, Spy49_1307c	1312813	High
operon_738	-	Spy49_1308c	1317209	High
operon_739	-	Spy49_1309c	1317777	High
operon_740	-	Spy49_1310c	1318118	Low
operon_741	-	Spy49_1311c	1320420	Low
operon_742	-	Spy49_1312c	1321689	High
operon_743	-	Spy49_1313c	1322482	Low
operon_744	-	Spy49_1314c	1323461	Low
operon_745	-	Spy49_1316c	1324788	High
operon_746	-	Spy49_1318c	1327249	High
operon_747	+	Spy49_1319	1328552	High
operon_748	-	Spy49_1320c, Spy49_1321c	1329195	High

operon_749	-	Spy49_1322c, Spy49_1323c, Spy49_1324c, Spy49_1325c, Spy49_1326c	1330779	High
operon_750	-	Spy49_1327c, Spy49_1328c, Spy49_1329c	1333409	Low
operon_751	+	Spy49_1330	1335995	High
operon_752	-	Spy49_1331c, Spy49_1332c, Spy49_1334c	1336954	High
operon_753	+	Spy49_1335	1340026	High
operon_754	-	Spy49_1336c	1341101	High
operon_755	-	Spy49_1337c, Spy49_1338c, Spy49_1339c, Spy49_1340c	1341506	High
operon_756	-	Spy49_1341c	1346387	Low
operon_757	-	Spy49_tRNAser1	1347108	Low
operon_758	-	Spy49_1342c, Spy49_1343c, Spy49_1344c, Spy49_1345c	1347265	High
operon_759	+	Spy49_1346, Spy49_1347	1350561	High
operon_760	-	Spy49_1348c, Spy49_1349c, Spy49_1350c	1351421	High
operon_761	-	Spy49_1351c	1353805	Low
operon_762	+	Spy49_1352, Spy49_1353	1355610	High
operon_763	+	Spy49_1354, Spy49_1355, Spy49_1356	1356661	High
operon_764	+	Spy49_1357	1359689	High
operon_765	+	Spy49_1358	1360190	High
operon_766	-	Spy49_1359c, Spy49_1360c, Spy49_1361c, Spy49_1362c, Spy49_1363c, Spy49_1364c, Spy49_1365c, Spy49_1366c, Spy49_1367c	1361948	High
operon_767	-	Spy49_1368c, Spy49_1369c, Spy49_1370c, Spy49_1371c	1369929	Low
operon_768	-	Spy49_1372c	1372792	High
operon_769	+	Spy49_1373	1373977	Low
operon_770	-	Spy49_1374c	1374222	Low
operon_771	-	Spy49_1375c, Spy49_1376c	1376143	Low
operon_772	-	Spy49_1377c, Spy49_1378c, Spy49_1379c	1377974	High
operon_773	-	Spy49_1380c	1380379	High
operon_774	-	Spy49_1381c	1380725	Low
operon_775	-	Spy49_1382c, Spy49_1383c, Spy49_1384c	1381048	Low
operon_776	-	Spy49_1385c	1384493	High
operon_777	-	Spy49_1386c	1384991	Low
operon_778	-	Spy49_1387c	1385932	High
operon_779	-	Spy49_1388c	1386840	Low
operon_780	+	Spy49_1389	1388353	High
operon_781	-	Spy49_1390c	1388901	High
operon_782	+	Spy49_1391	1390400	High
operon_783	-	Spy49_1392c, Spy49_1393c	1391444	High
operon_784	-	Spy49_1394c	1393578	High

operon_785	-	Spy49_1395c, Spy49_1396c, Spy49_1397c	1395858	Low
operon_786	-	Spy49_1398c, Spy49_1400c, Spy49_1401c, Spy49_1402c, Spy49_1403c	1398705	Low
operon_787	-	Spy49_1405c	1405827	Low
operon_788	-	Spy49_1407c	1410150	High
operon_789	-	Spy49_1408c, Spy49_1409c	1411786	Low
operon_790	-	Spy49_1410c	1413375	High
operon_791	-	Spy49_1720c	1416011	Low
operon_792	-	Spy49_1412c	1417301	High
operon_793	-	Spy49_1413c	1418366	Low
operon_794	-	Spy49_1414c	1419505	High
operon_795	-	Spy49_1415c	1422209	Low
operon_796	+	Spy49_1416, Spy49_1417	1424427	High
operon_797	-	Spy49_1418c, Spy49_1419c, Spy49_1420c	1426926	High
operon_798	-	Spy49_1421c, Spy49_1422c	1428451	Low
operon_799	-	Spy49_1423c	1430207	High
operon_800	+	Spy49_1424	1433156	High
operon_801	+	Spy49_1425	1434257	Low
operon_802	-	Spy49_1426c	1435099	High
operon_803	-	Spy49_1427c, Spy49_1428c	1435434	High
operon_804	-	Spy49_1429c	1436391	Low
operon_805	+	Spy49_1430	1436862	Low
operon_806	+	Spy49_1431	1438410	High
operon_807	-	Spy49_1432c, Spy49_1433c	1438897	Low
operon_808	-	Spy49_1434c	1439768	Low
operon_809	-	Spy49_1435c, Spy49_1436c	1442291	High
operon_810	+	Spy49_1437, Spy49_1438	1443328	High
operon_811	+	Spy49_1439	1444828	Low
operon_812	+	Spy49_1440	1447388	High
operon_813	-	Spy49_1441c	1447960	High
operon_814	+	Spy49_1442	1449260	High
operon_815	-	Spy49_1443c	1451686	Low
operon_816	-	Spy49_1444c	1452106	Low
operon_817	+	Spy49_1445	1452981	Low
operon_818	-	Spy49_1447c	1454040	High
operon_819	-	Spy49_1449c	1455418	High
operon_820	+	Spy49_1450	1457007	High
operon_821	+	Spy49_1451	1457762	Low
operon_822	-	Spy49_1452c	1460103	High
operon_823	+	Spy49_1453	1460507	High
operon_824	-	Spy49_1454c	1461229	High
operon_825	+	Spy49_1455	1461643	High

operon_826	+	Spy49_1456	1462652	Low
operon_827	-	Spy49_1457c, Spy49_1458c	1463202	High
operon_828	-	Spy49_1459c	1464727	High
operon_829	-	Spy49_1460c, Spy49_1461c, Spy49_1462c, Spy49_1463c, Spy49_1464c, Spy49_1465c, Spy49_1466c, Spy49_1467c, Spy49_1468c, Spy49_1470c, Spy49_1471c, Spy49_1472c, Spy49_1474c, Spy49_1475c, Spy49_1476c, Spy49_1477c, Spy49_1478c, Spy49_1479c, Spy49_1480c, Spy49_1481c	1466002	Low
operon_830	-	Spy49_1482c, Spy49_1483c	1481983	Low
operon_831	-	Spy49_1484c	1482683	High
operon_832	-	Spy49_1485c, Spy49_1486c, Spy49_1487c, Spy49_1488c	1482895	Low
operon_833	-	Spy49_1489c	1488362	High
operon_834	-	Spy49_1491c, Spy49_1492c, Spy49_1493c, Spy49_1494c	1489287	High
operon_835	-	Spy49_1496c, Spy49_1497c	1491134	Low
operon_836	-	Spy49_1498c, Spy49_1499c	1492074	Low
operon_837	-	Spy49_1500c, Spy49_1501c, Spy49_1502c	1493092	Low
operon_838	-	Spy49_1504c, Spy49_1505c	1494614	Low
operon_839	-	Spy49_1506c	1495452	Low
operon_840	-	Spy49_1507c	1497151	Low
operon_841	-	Spy49_1509c	1497865	High
operon_842	-	Spy49_1510c	1498507	High
operon_843	-	Spy49_1511c	1498768	Low
operon_844	-	Spy49_1512c, Spy49_1513c	1499108	Low
operon_845	-	Spy49_1514c	1500094	Low
operon_846	-	Spy49_1516c, Spy49_1517c, Spy49_1518c	1500547	High
operon_847	-	Spy49_1520c	1501979	High
operon_848	-	Spy49_1521c	1502394	Low
operon_849	+	Spy49_1522	1503377	High
operon_850	+	Spy49_1525	1503869	Low
operon_851	+	Spy49_1526	1504531	Low
operon_852	-	Spy49_1528c	1505484	Low
operon_853	+	Spy49_1529, Spy49_1530	1506006	High
operon_854	+	Spy49_1531	1507340	High
operon_855	+	Spy49_1532	1508554	Low
operon_856	-	Spy49_1533c	1508935	High
operon_857	-	Spy49_1534c	1509764	Low
operon_858	-	Spy49_1535c	1510451	High
operon_859	-	Spy49_1536c, Spy49_1537c	1511120	Low

operon_860	+	Spy49_1538	1512183	High
operon_861	-	Spy49_1540c, Spy49_1541c, Spy49_1542c	1513723	High
operon_862	+	Spy49_1543	1516613	Low
operon_863	+	Spy49_1544	1517630	High
operon_864	-	Spy49_1545c, Spy49_1546c, Spy49_1547c	1518085	High
operon_865	+	Spy49_1548	1520445	High
operon_866	+	Spy49_1549	1520799	Low
operon_867	-	Spy49_1550c, Spy49_1551c	1522613	High
operon_868	-	Spy49_1552c	1524480	Low
operon_869	-	Spy49_1553c	1525316	High
operon_870	-	Spy49_1554c	1526737	High
operon_871	-	Spy49_1555c	1527862	High
operon_872	-	Spy49_1556c, Spy49_1557c	1528925	High
operon_873	-	Spy49_1558c	1531111	High
operon_874	-	Spy49_1559c	1531660	High
operon_875	-	Spy49_tRNAleu7	1532737	High
operon_876	-	Spy49_1560c	1532829	High
operon_877	-	Spy49_1561c	1533926	Low
operon_878	-	Spy49_1562c	1535794	High
operon_879	-	Spy49_1563c	1536628	High
operon_880	+	Spy49_1564	1538107	Low
operon_881	-	Spy49_1565c, Spy49_1566c, Spy49_1567c, Spy49_1568c	1539145	Low
operon_882	-	Spy49_1569c	1542152	High
operon_883	-	Spy49_tRNAarg3	1542482	High
operon_884	-	Spy49_rRNA5s1	1542715	High
operon_885	-	Spy49_rRNA23s6	1542920	High
operon_886	-	Spy49_rRNA16s6	1546238	High
operon_887	-	Spy49_1571c	1547700	High
operon_888	+	Spy49_1572, Spy49_1573, Spy49_1574	1549457	Low
operon_889	-	Spy49_1575c	1555416	High
operon_890	-	Spy49_1576c, Spy49_1577c	1555805	Low
operon_891	-	Spy49_1578c, Spy49_1579c, Spy49_1580c, Spy49_1582c	1557974	Low
operon_892	-	Spy49_1583c	1562918	High
operon_893	-	Spy49_1584c	1563105	High
operon_894	-	Spy49_1585c, Spy49_1586c, Spy49_1587c, Spy49_1588c, Spy49_1589c, Spy49_1590c	1565073	Low
operon_895	+	Spy49_1591	1570498	High
operon_896	+	Spy49_1592, Spy49_1593	1571946	High
operon_897	+	Spy49_1594	1573062	High
operon_898	-	Spy49_1595c, Spy49_1596c	1573791	High

operon_899	-	Spy49_1597c, Spy49_1598c	1574794	Low
operon_900	-	Spy49_1599c	1575935	High
operon_901	-	Spy49_1600c, Spy49_1601c, Spy49_1602c	1576878	High
operon_902	-	Spy49_1603c, Spy49_1604c, Spy49_1605c	1579205	High
operon_903	-	Spy49_1606c, Spy49_1607c, Spy49_1608c	1581329	High
operon_904	-	Spy49_1609c, Spy49_1610c, Spy49_1611c, Spy49_1612c	1585414	High
operon_905	-	Spy49_1613c	1590246	High
operon_906	-	Spy49_1614c	1590633	Low
operon_907	+	Spy49_1615	1591292	High
operon_908	-	Spy49_1616c	1591956	High
operon_909	-	Spy49_1617c	1592575	Low
operon_910	-	Spy49_1618c	1593123	Low
operon_911	-	Spy49_1619c	1597819	High
operon_912	-	Spy49_1620c, Spy49_1621c, Spy49_1622c	1599785	Low
operon_913	-	Spy49_1624c	1602969	High
operon_914	-	Spy49_1625c	1603414	Low
operon_915	-	Spy49_1626c	1603890	Low
operon_916	-	Spy49_1627c	1607703	High
operon_917	-	Spy49_1628c	1609383	High
operon_918	-	Spy49_1629c	1610873	High
operon_919	+	Spy49_1630	1611982	High
operon_920	-	Spy49_1631c, Spy49_1632c	1613433	High
operon_921	-	Spy49_1633c	1616369	High
operon_922	+	Spy49_1634	1617982	High
operon_923	-	Spy49_1635c	1618785	High
operon_924	-	Spy49_1636c	1619783	High
operon_925	-	Spy49_1637c, Spy49_1638c, Spy49_1639c	1622275	High
operon_926	-	Spy49_1640c, Spy49_1641c, Spy49_1642c, Spy49_1643c	1625216	Low
operon_927	-	Spy49_1644c	1626803	Low
operon_928	-	Spy49_1646c, Spy49_1647c	1627613	Low
operon_929	-	Spy49_1649c	1629426	High
operon_930	+	Spy49_1651, Spy49_1652	1630131	High
operon_931	+	Spy49_1653, Spy49_1654, Spy49_1655	1630829	High
operon_932	+	Spy49_tRNAlys3	1634773	Low
operon_933	+	Spy49_1656, Spy49_1657	1634915	Low
operon_934	-	Spy49_1658c, Spy49_1659c	1636045	High
operon_935	+	Spy49_1660	1638088	High
operon_936	+	Spy49_1661, Spy49_1662, Spy49_1663, Spy49_1664	1639684	Low
operon_937	-	Spy49_1665c	1643049	High

operon_938	-	Spy49_1666c, Spy49_1667c	1643291	Low
operon_939	-	Spy49_1668c	1647049	High
operon_940	-	Spy49_1669c	1648536	Low
operon_941	-	Spy49_1670c	1652218	Low
operon_942	-	Spy49_1671c	1653672	High
operon_943	-	Spy49_1672c	1655042	High
operon_944	-	Spy49_1673c	1656467	High
operon_945	-	Spy49_1674c, Spy49_1675c	1658776	High
operon_946	-	Spy49_1676c, Spy49_1677c, Spy49_1678c, Spy49_1679c, Spy49_1680c	1660805	Low
operon_947	-	Spy49_1681c, Spy49_1682c	1666441	High
operon_948	-	Spy49_1683c	1667414	High
operon_949	-	Spy49_1684c	1669485	Low
operon_950	+	Spy49_1686	1673034	High
operon_951	-	Spy49_1687c	1673460	High
operon_952	-	Spy49_1688c	1674556	Low
operon_953	-	Spy49_1689c, Spy49_1690c	1674852	High
operon_954	+	Spy49_1691	1677332	High
operon_955	-	Spy49_1692c	1678492	High
operon_956	+	Spy49_1693	1679447	High
operon_957	+	Spy49_1694	1679682	High
operon_958	-	Spy49_1695c, Spy49_1696c, Spy49_1697c	1680276	High
operon_959	-	Spy49_1698c, Spy49_1699c, Spy49_1700c	1684725	High
operon_960	-	Spy49_1701c, Spy49_1702c	1686966	High
operon_961	+	Spy49_1703	1688846	High
operon_962	-	Spy49_1704c, Spy49_1705c	1689821	High
operon_963	-	Spy49_1706c	1690240	Low
operon_964	-	Spy49_1707c	1692620	Low
operon_965	+	Spy49_1708, Spy49_1709	1693454	High
operon_966	-	Spy49_1710c	1694641	High
operon_967	+	Spy49_1713	1696539	High
operon_968	-	Spy49_1714c	1698136	Low
operon_969	-	Spy49_1715c, Spy49_1716c	1699260	High
operon_970	-	Spy49_1717c, Spy49_1718c	1701438	High
operon_971	-	Spy49_1719c	1704488	High
operon_972	-	Spy49_tRNAcys1	1704947	High
operon_973	+	Spy49_1721, Spy49_1722	1707030	High
operon_974	-	Spy49_1723c	1709230	High
operon_975	+	Spy49_1724, Spy49_1725, Spy49_1726, Spy49_1727, Spy49_1728	1710759	High
operon_976	+	Spy49_1729, Spy49_1730	1716861	Low
operon_977	+	Spy49_1731	1720037	High

operon_978	-	Spy49_1732c	1721062	High
operon_979	+	Spy49_1733	1724331	High
operon_980	+	Spy49_1734	1725283	Low
operon_981	-	Spy49_1735c	1726508	High
operon_982	-	Spy49_1736c	1728555	Low
operon_983	-	Spy49_1737c	1730321	Low
operon_984	+	Spy49_1738	1732553	High
operon_985	-	Spy49_1739c	1733538	High
operon_986	+	Spy49_1740	1734089	High
operon_987	+	Spy49_1742	1734983	Low
operon_988	-	Spy49_1743c, Spy49_1744c, Spy49_1745c, Spy49_1746c	1735746	High
operon_989	-	Spy49_1747c	1738287	High
operon_990	-	Spy49_1748c	1740599	High
operon_991	-	Spy49_1749c, Spy49_1750c, Spy49_1751c	1742580	High
operon_992	-	Spy49_1752c	1743708	High
operon_993	-	Spy49_1753c	1744394	High
operon_994	-	Spy49_1754c	1745611	Low
operon_995	-	Spy49_1755c, Spy49_1756c, Spy49_1757c, Spy49_1758c	1747037	High
operon_996	-	Spy49_1759c	1751455	High
operon_997	-	Spy49_1761c	1754203	Low
operon_998	+	Spy49_1762	1755076	High
operon_999	+	Spy49_1763	1756834	Low
operon_1000	-	Spy49_1764c, Spy49_1765c, Spy49_1767c, Spy49_1768c	1757214	High
operon_1001	-	Spy49_1769c	1761877	Low
operon_1002	+	Spy49_1770	1763499	High
operon_1003	+	Spy49_1771	1763736	Low
operon_1004	-	Spy49_1772c	1763913	Low
operon_1005	+	Spy49_1773, Spy49_1774, Spy49_1775, Spy49_1777, Spy49_1778	1764056	Low
operon_1006	+	Spy49_1779	1767837	High
operon_1007	-	Spy49_1780c	1769589	Low
operon_1008	+	Spy49_1781, Spy49_1782, Spy49_1783	1770449	High
operon_1009	-	Spy49_1784c	1772555	High
operon_1010	+	Spy49_1785	1774918	High
operon_1011	-	Spy49_1786c	1775628	High
operon_1012	-	Spy49_1787c, Spy49_1788c, Spy49_1789c, Spy49_1790c	1776422	Low
operon_1013	-	Spy49_1791c	1781164	High
operon_1014	-	Spy49_1792c	1783162	Low

operon_1015	-	Spy49_1793c	1783693	High
operon_1016	+	Spy49_1794, Spy49_1795	1785718	High
operon_1017	-	Spy49_1796c	1787678	High
operon_1018	-	Spy49_1797c, Spy49_1798c, Spy49_1799c, Spy49_1800c, Spy49_1801c, Spy49_1802c, Spy49_1803c	1788829	High
operon_1019	+	Spy49_1804, Spy49_1806	1795589	High
operon_1020	+	Spy49_1808	1798342	Low
operon_1021	+	Spy49_1809, Spy49_1810	1799634	High
operon_1022	-	Spy49_1811c	1801205	High
operon_1023	-	Spy49_1812c	1802261	High
operon_1024	-	Spy49_1813c	1803933	Low
operon_1025	+	Spy49_1814	1805503	High
operon_1026	+	Spy49_1815	1806440	Low
operon_1027	+	Spy49_1817	1808141	High
operon_1028	-	Spy49_tRNAasn3	1811245	Low
operon_1029	-	Spy49_tRNAglu1, Spy49_tRNAglu3	1811349	Low
operon_1030	+	Spy49_tRNAarg7	1812779	High
operon_1031	-	Spy49_1820c	1812870	High
operon_1032	+	Spy49_1821	1813637	High
operon_1033	+	Spy49_1822	1814949	Low

RightBound	RightBound Confidence
1664	Low
2888	High
3452	Low
4629	High
8820	Low
9599	Low
14799	High
16622	High
18609	High
21942	High
22110	High
22327	Low
22516	Low
23075	Low
24607	High
25164	High
27944	High
28107	High
28326	Low
29492	Low
29771	High
32370	High
33588	High
34572	Low
35909	Low
40687	Low
43392	Low
44122	Low
45759	High
47025	Low

48541 Low
50342 Low
51961 Low
53346 High
54614 Low
55683 Low
58418 High
61360 High
62680 High
64273 High
64912 Low

67527 Low

71386 Low
72207 Low
72704 Low
73930 Low
75807 High
76636 Low

79315 High
81590 High
81726 Low
84927 High
85141 Low
85228 Low
85307 Low

86130 High
86225 High
87879 Low
89973 High
90417 Low
91720 High
94231 Low
97988 Low

101673	High
102208	High
103257	Low
105984	Low
106969	Low
108189	High
108697	Low
112298	High
113738	High
114190	High
117056	Low
118676	High
124313	Low
125736	High
129503	High
130422	High
132193	Low
133189	High
135862	Low
136591	High
138187	High
143494	Low
147512	High
149761	High
151623	Low
152848	High
153557	High
157584	High
158024	High
158638	High
159257	High
159579	Low
161290	High
164110	High
166161	Low
169090	High
171032	High
172372	High

175814	High
178642	Low
179783	Low
180271	Low
182062	Low
182506	Low
183105	Low
184525	Low
186508	High
187514	High
188808	High
190181	High
191255	High
192782	High
194507	High
194848	Low
195620	High
197545	High
199548	High
200711	High
202700	High
206923	High
207815	High
209890	Low
210498	High
211414	Low
213019	High
217159	High
217607	Low
219343	High
219823	High
222117	High
224071	Low
226725	Low
227736	Low
229305	Low
230256	High
235087	High
236132	Low
238725	High
240041	Low

242324	High
243691	High
244119	Low
246459	High
248454	Low
249503	High
251560	High
257052	High
258290	High
259692	High
266506	High
267041	High
269002	High
269452	High
272320	High
272761	High
273560	High
278446	Low
280080	Low
280884	High
281914	High
283203	Low
285112	Low
286646	Low
287972	High
290089	High
290881	High
292518	High
293359	Low
294421	High
295856	Low
299934	High
303415	Low
305998	Low
307638	Low
308194	High

310091	High
311100	Low
312301	High
312832	Low
317712	Low
318810	High
319490	High
320370	Low
320921	High
322882	Low
324330	High
325218	Low
326239	High
326955	High
330876	High
332453	High
334202	High
335093	Low
335811	High
336687	Low
338513	Low
338886	Low
340624	High
341296	High
341971	High
342880	High
344288	High
349604	High
351057	Low
353231	High
357411	High
358378	High
359491	High
359895	Low
360815	High
361517	High
362814	High
363659	Low
365053	High

365370	High
366418	High
367110	High
368659	Low
369224	Low
369566	High
372361	Low
372941	Low
373751	High
374470	High
376781	High
379490	Low
381080	Low
384175	Low
384639	High
385351	High
386417	High
388142	High
389129	High
391641	High
392050	High
392778	Low
393570	High
395197	High
396123	High
397054	Low
398253	High
400343	High
402239	Low
403263	Low
404792	High
405387	High
406268	High
407004	Low
407918	Low
408662	Low
409107	Low
411032	High
412606	High
413624	Low
415243	Low

416880	Low
418312	Low
418606	Low
422191	High
424625	High
425849	High
428089	High
429303	High
431933	High
434100	High
436890	High
437368	Low
439998	Low
441088	Low
444079	High
448713	High
449845	High
455663	Low
456914	Low
459813	High
462940	Low
463151	Low
463537	Low
464126	High
465070	Low
465421	Low
466536	Low
467977	Low
468895	Low
470063	High
471555	High
471645	Low
474932	High
476349	High
480866	High
482957	High
485716	High
486159	High
489010	High

489296	Low
490607	Low
491968	High
492418	High
492689	Low
494246	High
495343	High
496216	Low
496909	High
497654	High
498799	Low
499857	Low
502173	High
505193	Low
506159	High
508159	High
509114	High
512503	High
514221	High
514776	Low
517623	High
518702	High
520696	Low
524526	High
527977	High
529269	Low
530834	Low
533590	High
534627	Low
535505	High
538258	Low
540991	High
544177	High
545722	High
547490	High
549554	High
549893	High
551067	High
551746	High
553498	Low

553956	High
554582	High
555516	Low
557652	Low
559626	Low
560356	Low
561832	High
563282	Low
565040	High
568963	High
570092	High
578148	High
581539	Low
582199	High
585412	Low
590253	Low
592203	High
594204	High
596224	Low
598751	High
601440	High
602459	High
609513	Low
610132	High
610745	High
611227	High
614388	Low
614913	Low
615838	High
625378	Low
629239	High
631101	Low
631316	Low
632534	Low
633940	High

636367	High
640006	High
641579	High
643447	High
646096	Low
647258	Low
648526	High
651201	High
655532	Low
658837	High
662380	Low
664244	High
664921	High
669327	High
670355	Low
671087	Low
673514	Low
675614	High
679487	Low
680184	Low
681029	High
681583	Low
682954	High
685217	High
688374	High
689630	Low
691903	Low
694538	High
698720	High
701239	High
705008	High
705878	High
708172	High
711911	Low
712949	High
713766	Low
714893	Low

715635 High
717224 Low
718835 Low
720456 High
721288 Low
722612 High
723311 High
727957 High
729108 High
729421 High
729660 Low
731010 High
731203 High
731801 High
732983 High
733589 Low
734927 High
735192 High

739179 Low
742114 Low
744160 Low
746841 Low
748447 Low
749615 High
750885 High
752124 High
752455 Low
753282 Low

754712 Low
754986 High

761525 High
763520 Low
763941 Low
764434 High

769299 Low

773339 Low

784892	Low
785720	High
786259	Low
787447	High
790639	High
792362	High
793948	Low
795670	High
797524	High
798805	High
800755	High
803155	Low
804820	Low
806834	High
809955	Low
811106	High
813294	High
816637	High
820772	High
827190	Low
828162	High
830196	Low
831472	High
832121	Low
833216	High
834409	Low
836029	High
839724	Low
843127	Low
843885	Low
845374	High
846797	High
848110	High
849581	High
850638	High
854581	High

859388	High
861625	High
864372	Low
865479	High
866440	High
868195	Low
868980	Low
870176	High
870927	Low
871528	Low
872403	Low
875046	High
875709	High
878162	Low
879232	High
880137	Low
881136	Low
883598	High
884876	Low
885157	Low
886973	High
888275	High

898215	High
899893	High
901049	High
904460	High
905180	Low
906041	High
907093	Low
908186	High
910021	High
910998	Low
913160	High
913986	Low
915707	High
917207	High
918690	High

922185 Low
923878 High
925432 High
926366 Low
927825 Low

932690 High
933874 Low
934697 Low
936193 High
937325 High
938969 Low
939362 Low
940124 High
941742 Low
943375 High
944856 Low
946540 High
948039 High
949795 High
950233 High
953940 Low

960179 High
961620 Low
962292 Low
964150 High
967913 High
969859 High
970804 High
972012 Low
972356 High
974707 Low
977357 Low

982510 High
984959 Low
985393 High
987320 Low
988731 High

993844	High
994720	Low
995382	Low
998043	High
1000532	High
1002408	Low
1003799	High
1004917	High
1007364	High
1007806	High
1009876	High
1010541	High
1013345	High
1016534	High
1018583	High
1019377	Low
1019763	High
1020691	High
1024833	Low
1025904	High
1027382	High
1029827	High
1031177	High
1031323	Low
1037036	High
1039229	High
1042361	High
1044081	Low
1047335	Low
1049907	High
1050814	Low
1052558	High
1053176	High
1054133	Low
1055028	High
1056604	Low
1058691	Low
1058917	Low

1059831	High
1060496	High
1060996	High
1062491	High
1064224	High
1064543	Low
1065478	Low
1067106	Low
1068682	Low
1070609	Low
1074356	High
1075211	High
1076784	High
1078509	High
1081294	Low
1082182	Low
1084853	Low
1085811	High
1086633	High
1087840	High
1089203	High
1090725	High
1092952	High
1093513	Low
1096983	High
1098331	Low
1098567	High
1099604	Low
1101572	High
1104510	Low
1105780	High
1106528	High
1108109	High
1109987	High
1111096	Low
1112020	Low
1112805	High
1115246	High
1116542	Low
1117216	Low
1118301	High

1121417	Low
1122218	High
1123367	High
1125415	High
1127288	Low
1129184	High
1131431	High
1134093	Low
1134940	High
1135579	High
1136355	Low
1137101	High
1138265	Low
1140429	High
1141041	High
1143368	High
1144614	Low
1147868	Low
1150104	High
1151077	High
1153046	High
1154592	High
1156147	High
1156506	High
1159079	High
1160035	High
1163133	Low
1169271	High
1173025	High
1173676	Low
1175592	High
1177134	Low
1177331	High
1178170	High
1178896	Low
1180871	Low
1182434	Low
1184592	Low
1185808	Low
1189652	Low

1192818	High
1193726	High
1194400	High
1198885	High
1201582	High
1207641	Low
1210250	Low
1212918	Low
1213865	High
1214779	High
1215008	Low
1217336	High
1219193	High
1220182	Low
1227602	High
1231236	High
1232836	Low
1237144	High
1241285	High
1243071	Low
1244830	High
1245690	Low
1246527	High
1247270	High
1247568	High
1247765	High
1248002	High
1248161	Low
1250969	High
1251494	High
1253083	High
1254582	High
1256645	Low
1257341	Low
1258357	High
1262509	High

1267552	High
1270055	Low
1271042	High
1272783	High
1273871	Low
1278744	Low
1279356	High
1282079	Low
1282489	High
1282859	High
1283502	Low
1284138	Low
1286403	High
1288179	High
1290580	High
1292200	Low
1293233	Low
1293559	High
1295152	High
1296756	Low
1301380	High
1303980	High
1306880	Low
1308927	High
1309824	High
1312707	High
1316934	High
1317630	High
1318117	Low
1320419	Low
1321688	Low
1322481	Low
1323434	High
1324686	High
1327248	Low
1328521	High
1329161	High
1330660	High

1333408	Low
1335849	High
1336839	High
1339900	High
1341050	High
1341500	Low
1346386	Low
1347050	High
1347264	Low
1350540	High
1351420	Low
1353718	High
1355609	Low
1356436	High
1359601	High
1360189	Low
1361734	High
1369928	Low
1372664	High
1373976	Low
1374221	Low
1376142	Low
1377894	High
1380001	High
1380724	Low
1381047	Low
1384349	High
1384990	Low
1385833	High
1386839	Low
1388175	High
1388880	High
1390375	High
1391419	High
1393572	Low
1395857	Low

1398704 Low

1405826 Low
1409792 High
1411780 High
1413372 Low
1415942 High
1417229 High
1418360 High
1419491 Low
1422164 High
1424303 High
1426902 High
1428446 High
1430137 High
1433074 High
1434256 Low
1435066 High
1435280 Low
1436389 High
1436857 High
1438409 Low
1438896 Low
1439767 Low
1442284 Low
1443168 High
1444827 High
1447385 Low
1447933 High
1449114 High
1451679 High
1452105 Low
1452980 Low
1454039 Low
1455264 High
1456944 High
1457761 Low
1460062 High
1460312 High
1461146 High
1461635 Low
1462651 Low

1463190	High
1464619	High
1466001	Low
1481974	High
1482675	Low
1482936	High
1487749	High
1489195	High
1491133	Low
1492073	Low
1493091	Low
1494613	Low
1495451	Low
1497150	Low
1497690	Low
1498358	High
1498717	High
1499107	Low
1500093	High
1500536	Low
1501915	Low
1502393	Low
1503319	Low
1503736	High
1504357	High
1505483	High
1505712	High
1507309	High
1508553	Low
1508882	High
1509763	Low
1510448	Low
1511119	Low
1512182	High

1513696	High
1516612	Low
1517626	Low
1518051	High
1520299	High
1520798	Low
1522571	High
1524478	High
1525083	High
1526565	High
1527591	High
1528757	High
1530977	High
1531356	High
1532610	High
1532828	Low
1533925	Low
1535792	Low
1536468	High
1537971	High
1539144	Low
1541713	High
1542437	High
1542516	High
1542818	High
1546188	High
1547660	High
1549456	Low
1555308	High
1555804	Low
1557973	Low
1562913	Low
1563044	High
1565072	Low
1570175	High
1571568	High
1572586	High
1573576	High
1574790	High

1575933	Low
1576835	High
1579034	High
1581327	Low
1585233	High
1589937	High
1590628	High
1591125	High
1591942	High
1592574	Low
1593122	Low
1597582	High
1599784	Low
1602793	High
1603413	Low
1603889	Low
1607482	High
1609333	Low
1610597	High
1611701	High
1613385	High
1616131	High
1617689	High
1618763	High
1619779	Low
1622080	High
1625215	Low
1626794	High
1627612	Low
1629057	High
1630007	High
1630747	High
1634772	High
1634862	High
1636020	High
1637169	High
1639682	High
1643031	High
1643290	High

1646855	High
1648535	Low
1652217	Low
1653538	High
1654911	High
1656397	High
1658192	High
1660804	Low
1666439	Low
1667315	High
1669484	Low
1672695	High
1673366	High
1674542	High
1674792	High
1676619	High
1678295	High
1679370	High
1679677	Low
1680222	High
1684561	High
1686797	High
1688739	High
1689813	High
1690219	High
1692619	Low
1693046	High
1694590	High
1696353	High
1698133	High
1699182	High
1701314	High
1704394	High
1704807	High
1706618	High
1709194	High
1710621	High
1716860	Low
1719960	Low
1721039	High

1724163 High
1725282 Low
1726430 High
1728554 Low
1730320 Low
1732466 High
1733311 High
1733962 Low
1734982 Low
1735707 High

1738262 High
1740542 High
1742371 High
1743633 High
1744135 High
1745584 High
1746919 High

1751357 High
1754202 Low
1754824 High
1756833 Low
1757190 High

1761876 Low
1763344 High
1763735 Low
1763912 Low
1764055 High

1767541 High
1769588 High
1770292 High
1772519 High
1774903 Low
1775585 Low
1776418 High

1781111 High
1783161 Low
1783691 Low

1785578	High
1787650	High
1788336	High
1795320	High
1798341	Low
1799334	High
1801190	High
1802110	High
1803932	Low
1805318	High
1806439	Low
1808134	Low
1811211	High
1811348	High
1811410	Low
1812860	Low
1813481	High
1814936	High
1815784	Low
